# The Samata intervention to increase secondary school completion and reduce child marriage among adolescent girls: results from a cluster-randomised control trial in India

**DOI:** 10.7189/jogh.09.010430

**Published:** 2019-06

**Authors:** Ravi Prakash, Tara S Beattie, Prakash Javalkar, Parinita Bhattacharjee, Satyanarayana Ramanaik, Raghavendra Thalinja, Srikanta Murthy, Calum Davey, Mitzy Gafos, James Blanchard, Charlotte Watts, Martine Collumbien, Stephen Moses, Lori Heise, Shajy Isac

**Affiliations:** 1 Karnataka Health Promotion Trust (KHPT), Rajajinagar, Bangalore, India; 2 London School of Hygiene & Tropical Medicine (LSHTM), Departments of Global Health and Development and Public Health, Environments and Society, London, UK; 3 University of Manitoba, Departments of Community Medicine and Medical Microbiology, Winnipeg, Canada; 4 Johns Hopkins Bloomberg School of Public Health and JHU School of Nursing, Department of Population, Family and Reproductive Health, Baltimore, Maryland, USA; * Joint first authorship; ** Joint last authorship

## Abstract

**Background:**

Secondary education and delayed marriage provide long-term socio-economic and health benefits to adolescent girls. We tested whether a structural and norms-based intervention, which worked with adolescent girls, their families, communities, and secondary schools to address poverty, schooling quality and gender norms, could reduce secondary school drop-out and child marriage among scheduled-caste/scheduled-tribe (SC/ST) adolescent girls in rural settings of southern India.

**Methods:**

80 of 121 villages in Vijayapura and Bagalkote districts, Karnataka State, were randomly selected (control = 40; intervention = 40). All 12-13 year-old SC/ST girls in final year of primary school (standard 7^th^) were enrolled and followed for 3 years (2014-2017) until the end of secondary school (standard 10^th^). Primary trial outcomes were proportion of girls who completed secondary school and were married, by trial end-line (15-16 years). Analyses were intention-to-treat and used individual-level girl data.

**Results:**

92.6% (2275/2457) girls at baseline and 72.8% (1788/2457) at end-line were interviewed. At end-line, one-fourth had not completed secondary school (control = 24.9%; intervention = 25.4%), and one in ten reported being married (control = 9.6%; intervention = 10.1%). These were lower than expected based on district-level data available before the trial, with no difference between these, or other schooling or sexual and reproductive outcomes, by trial arm. There was a small but significant increase in secondary school entry (adjusted odds ratio AOR = 3.58, 95% confidence interval CI = 1.36-9.44) and completion (AOR=1.54, 95%CI = 1.02-2.34) in Vijayapura district. The sensitivity and attrition analyses did not impact the overall result indicating that attrition of girls at end-line was random without much bearing on overall result.

**Conclusions:**

Samata intervention had no overall impact, however, it added value in one of the two implementation districts- increasing secondary school entry and completion. Lower than expected school drop-out and child marriage rates at end-line reflect strong secular changes, likely due to large-scale government initiatives to keep girls in school and delay marriage. Although government programmes may be sufficient to reach most girls in these settings, a substantial proportion of SC/ST girls remain at-risk of early marriage and school drop-out, and require targeted programming. Addressing multiple forms of clustered disadvantage among hardest to reach will be key to ensuring India “leaves no-one behind” and achieves its gender, health and education Sustainable Development Goal aspirations.

**Trial registration:**

ClinicalTrials.gov registration number NCT01996241.

In 42 low-middle income countries (LMICs), 36% of girls are married before their 18^th^ birthday [1]. Girls are more likely to experience child marriage (<18 years) than boys, and rates are highest among the poorest and most socially disadvantaged [1]. Early marriage is associated with a range of adverse outcomes, including inadequate access and underuse of health services, early child-bearing and low schooling and literacy levels [2,3]. Effects continue into the next generation, with children (especially girls) born to early married mothers less likely to ever into or complete primary school education [4]. In contexts such as India, where fertility is strongly tied to marriage, first pregnancy and childbirth often follow quickly after marriage. Early child-bearing (<18 years) increases the risk of delivery complications and is associated with increased maternal mortality, infant mortality and low birth weight; children of young mothers are also more likely to experience child stunting, malnutrition and anaemia [2,5,6]. The global economic costs associated with the impacts of child marriage on fertility and population growth, children’s health, and education are high [7].

In India, traditionally girl child marriage has strong social and economic implications with marriages arranged between families anytime from birth through adolescence. Girls’ families are expected to pay substantial dowries and all wedding-related costs [8]. As a result, girls are traditionally seen as an economic burden for families and preference for sons is high [8]. Concerns around maintaining a girl’s ‘purity’ until marriage are common and are connected with norms of family ‘honour’ [8]. Traditionally, this leads to a girl’s mobility being restricted once she reaches menarche and can lead to premature withdrawal from school and early marriage. A girl’s purity after marriage is also important, but marriage transfers the responsibility of protecting her purity from the natal to the spouse’s home. Harassment by men and boys (name calling, whistling, being pelted with small stones), is commonly described in Indian settings as *“eve-teasing”* [8]. Although the victims may not experience serious harm, the importance of maintaining a girl’s sexual purity means fear of eve teasing can lead to a family terminating a girl’s education and arranging an early marriage. Following marriage, girls usually move from their natal to their spouse’s family home, where they assume the traditional role of wife, mother, and homemaker.

Education is a powerful determinant of adolescent health and driver of socio-economic progress [1,9,10]. Secondary education is associated with reduced teenage births and older age at marriage [10]. Although primary education is now widespread in many LMIC [11], many forces operate to exclude or divert adolescents – particularly girls - from secondary education, including poor academic achievement, the costs of education, the need for adolescent labour especially in rural areas, lack of parental aspirations for education, marriage, pregnancy, and maternal migration [12-14]. Similar to child marriage, rates of school drop-out are highest among girls, particularly among the poorest and most socially disadvantaged, rural families [9,14].

In northern Karnataka, India, where this study took place, the *Devadasi* tradition of sex work is practiced, whereby girls from *Devadasi* families are dedicated to the Temple goddess Yellemma, and upon reaching menarche, commence socially sanctioned sex work [15]. Previous work in north Karnataka has identified high levels of HIV (>30%) among female sex workers (FSWs) and migration of young adolescent girls to major cities in the neighbouring State of Maharashtra, to be inducted into sex work, with a high risk of HIV infection [16]. The current study was designed to address the “up-steam structural drivers” of HIV infection among girls and women from *Devadasi* and other sex work families living in this region. Almost all *Devadasi* belong to SC/ST communities. Scheduled caste/scheduled tribe (SC/ST) are legal caste categories in India and refer to indigenous populations from the most disadvantaged strata of Indian society. Some members of this group prefer the term ‘Dalit’ which connotes people who are ‘broken, crushed and torn apart’ and which is part of a wider political vision. However, to keep with the project terminology, we use the term ‘SC/ST’ in this article. SC/ST girls living in northern Karnataka have the lowest rates of secondary school enrolment and retention, and the highest rates of child marriage in the state, highlighting gender, caste and regional disparities in educational uptake and child marriage [17,18]. A central hypothesis of the intervention and evaluation was that keeping SC/ST girls in secondary school would delay their age at marriage and delay girls from *Devadasi* families entering into sex work. This, in turn, would reduce HIV-incidence among adolescent girls and young women entering sex work.

There have been four recent systematic and two comprehensive reviews of child marriage and schooling interventions with adolescent girls. Additionally, we searched Medline and ERIC from Jan 1, 2016 to April 1, 2018, and searched websites of various non-governmental organisations working on child marriage and school drop-out (3ie, UNICEF, Population Council, ICRW, Save The Children). In Medline we did keyword searches using the terms “child marriage”, or “school”, and the clinical trial filter option. We identified sixteen trials of interventions which sought to reduce child marriage or increase secondary school enrolment, attendance or completion in eleven low and middle income countries (LMIC) – Mexico, Colombia, South Africa, Zimbabwe (n = 2), Malawi, Kenya (n = 3), Tanzania, Uganda, Nepal, India (n = 2), Bangladesh (n = 2); eight were published before the start of our trial (2000-2012) and eight since (2013-2018) [19-34]. Most trials (11/16) used an economic component (cash transfers, school subsidies, economic support, improving livelihood options for unmarried, educated girls) as a key part of the intervention [20,21,24-29,32,34,35]; 10/11 trials with an economic component reported significant improvements in secondary school enrolment, attendance, or completion and 5/7 trials with an economic component reported significant reductions in child marriage. Among the five trials which used other intervention designs [19,22,23,30,33], two reported significant improvements: a community-based skills-development programmes for girls in Bangladesh reported significant increases in school attendance and significant reductions in child marriage and an adolescent development and empowerment programme in Uganda found no difference in school enrolment but significant reductions in marriage and fertility outcomes [19,22]. Conversely, an adolescent development and empowerment programme with a micro-credit component in Tanzania, a menstrual cup provision intervention in Nepal and a parent and community engagement programme in west India all found no difference in schooling and/or marriage outcomes [23,30,33].

We report results of the Samata intervention, developed as part of the STRIVE research programme consortium, a UKAid-funded programme of research and action devoted to tackling the structural drivers of HIV. A key tenet of the Theory of Change was that keeping SC/ST adolescent girls in secondary school would delay the age at marriage and delay girls born into the *Devadasi* system from entering into sex work. Moving away from cash transfer intervention design to a more sustainable model, this was the first trial globally designed to address normative and structural factors hypothesized to encourage school dropout and early marriage.

## METHODS

### Setting

The Samata study took place in Vijayapura and Bagalkote districts, Karnataka state, southern India, among SC/ST adolescent girls living in rural villages from March 2014 to September 2017. The two districts are demographically similar, with around three quarters of the population living in rural areas, and 85% of rural SC/ST households living below the poverty line [36,37].

### Participants

**Figure 1:**
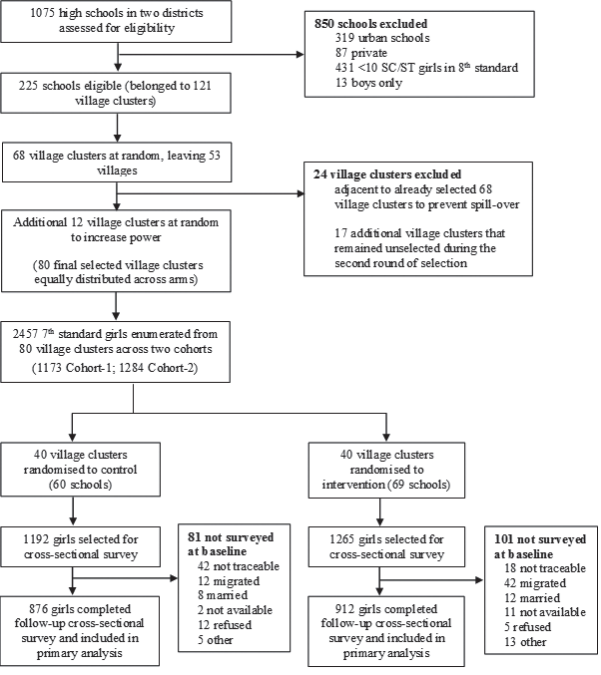
Flow of the participants in the trial.

All SC/ST girls living in the study villages and enrolled in standard 7 (the final year of primary school) in 2012 and 2013, were eligible for the study and were recruited in two cohort waves, one academic year apart (**Figure 1**). Cross-sectional baseline surveys were conducted in Feb-Jun 2014 (cohort 1) when girls were in their final term of the first year of secondary school, and Jul-Sep 2014 (cohort 2), when girls were in their first term of secondary school. The intervention was delivered to all SC/ST girls aged 13-16 years in the intervention villages, regardless of whether they were enrolled in the study. Although girls were expected to receive 36 months of intervention activities, due to funding issues at the start of the trial, cohort 1 girls received 18 months of intervention exposure (starting when they were in year 9) and cohort 2 girls received 30 months of intervention exposure (starting when they were in year 8) (Figure S1 in **Online Supplementary Document**). End line surveys were conducted in May-Jul 2016 (cohort 1) and May-Jul 2017 (cohort 2), after their secondary school final exams. Parents/guardians provided written informed consent, and adolescent girls provided written informed assent, for their participation in the study. The surveys were administered by trained female field investigators and all interviews were conducted in *Kannada* (the local language) in a private setting. Repeat visits were made to find girls who were not available on the survey day. Interviewers referred girls needing help or counselling support to the nearest *Santhwana* Centre (Government-funded centres providing financial and emotional support to women experiencing violence, forced marriage, and other gender-based issues). The study was approved by ethics committees at St John’s Medical College, Bangalore (Ref 111/2013), LSHTM (Ref 7083), and the University of Manitoba (Ref H2014:414). The study protocol was registered at clinicaltrials.gov (NCT01996241) [18].

### Intervention

The Samata intervention was a multi-level intervention designed to address normative and structural factors hypothesized to encourage school dropout and early marriage in this setting. A theory of change was developed with programme and research study team members and key stakeholders, which posited that poverty, gender norms, poor accountability of schools and communities, and poor education, perpetuate traditions of underage marriage and sex work that heighten vulnerability to HIV infection and diminish the quality of life for adolescent girls from SC/ST girls in rural northern Karnataka. The theory of change informing the project’s design is available in Figure S2 in **Online Supplementary Document**. The intervention components were designed to overcome the existing barriers of poverty and low value for girl education, together with tradition and existing gender norms which encourage families to remove their daughters from school, marry daughters early or dedicate them as *Devadasi* sex workers, and allow boys to be disrespectful towards girls and devalue girls’ education. They were also designed to enable schools and community groups to pro-actively track and address the needs of girls to improve their retention in school and to provide girls with role models, aspirations for professional careers and a sense of solidarity with other girls. A description of the programme can be seen in Appendix S1 in **Online Supplementary Document** [18]. The programme worked with adolescent girls and boys, parents and village communities to change norms around girl education and marriage and overcome poverty-related drivers of school drop-out and early marriage by (i) establishing safe spaces for girls to meet and develop life skills, (ii) providing academic tutoring for girls, (iii) forming boys sports groups to sensitise boys to appreciate girls’ rights and treat girls respectively, (iv) sensitising parents to value girls education and challenging norms around age of girl child marriage, and (v) linking families to government financial incentives for girls in school. The intervention also worked with schools to introduce girl tracking systems, introduce school safety policies, train school staff on issues around gender and girl school drop-out, and strengthen the school governing committees. The programme was delivered to all SC/ST girls aged 13-16 years in the intervention village clusters, regardless of if they were enrolled in the study.

### Design

The evaluation design involved a two-arm cluster-randomised controlled trial with parallel assignment (**Figure 1**), using a mixed-method approach; baseline and endline quantitative surveys, a longitudinal qualitative study, and process evaluation. From a sample frame of 121 villages, we randomly selected 80 village clusters (40 intervention, 40 control), encompassing 296 villages (119 intervention, 177 control) and 129 secondary schools (69 intervention, 60 control) [18]. A “village cluster” comprises one “main” village with one or more eligible high-schools plus the surrounding ‘feeder’ villages, which do not contain a high-school but have SC/ST adolescents living there who attend the high-school in the “main” village.

We estimated the sample size based on the prevalence of the selected outcomes using available data from the district and state-wide surveys. Four outcomes were considered, i). proportion of girls transitioning from 7^th^ to 8^th^ standard, ii). proportion completing standard 10^th^, iii). proportion married before age 15 years, and iv). proportion of girls reported sexual intercourse before age 15. We used a varying effect size for different outcomes based on the existing data and past trends and with an anticipation of a minimum detectable effect size of 20%-25% for secondary school completion and 25%-30% for child marriage and early sexual debut [15]. Due to the uncertainty about several underlying parameters of the sample size calculation, especially the between village variation (κ) in the outcomes, we allowed for a range of calculations for κ depending on the relationship between the number of cluster and value of κ to achieve a realistic estimate for different parameters. After applying all possible combinations, selection of 80 village clusters with a harmonic average of 30 SC/ST girls per cluster, was calculated to be sufficient to detect the effect estimates for the outcomes above, with 80% power and 5% statistical significance. With a conservative estimate of loss-to-follow-up and assumption of at least 28 low caste girls per cluster, we estimated to detect 33% reduction in the risk of not transitioning between 7^th^ to 8^th^ standard, and 25% reduction in the risk of not retaining in school until the completion of 10^th^ standard [15].

### Outcomes

The two primary outcomes were (i) completion of secondary school [sit 10^th^ standard exam] and (ii) marriage [by trial end line]. Four secondary outcomes were (i) entering into secondary school [start 8^th^ standard]; (ii) passing end of secondary school exams [pass 10^th^ standard exam]; (iii) sexual debut [by trial end line] and (iv) marriage *and* co-habitation with husband [by trial end line]. The primary and secondary trial outcomes were amended and registered at the clinical trials registry during the trial because (i) personal communication with experts in cluster-RCT design recommended reducing the number of primary outcomes from four to two, (ii) qualitative findings revealed that the first night ceremony (sexual debut) and co-habitation with a spouse may happen months after marriage, (iii) baseline surveys showed we had insufficient girls from *Devadasi* families to measure sex work entry as an outcome, and (iv) insufficient girls were married at baseline to have pregnancy at endline as an outcome. Schooling and marriage outcomes were measured using a face-to-face questionnaire; sexual debut was measured using a self-completed questionnaire (after the face-to-face questionnaire).

### Statistical analysis

The trial was not masked. The statistician (RP) and the research study team were masked to allocation while performing and interpreting analyses. We did an intention-to-treat analysis using data from our cross-sectional follow-up surveys; data was pooled for cohort 1 and cohort 2 for all primary and secondary outcomes, except for secondary outcome (i) entering into secondary school, for which we used baseline data from cohort 2 (as cohort one were already in the second year of secondary school when the intervention activities began). All data were collected on paper and double entered using the Census and Survey Processing System (CSPro; https://www.census.gov/population/internatioan/software/cspro). All analyses were done in STATA (version 14.0; StataCorp, College Station, TX, USA).

All trial outcomes were measured at the level of individual girl participants. Cluster-level summaries were also reported. The analysis was done using individual-level girl data, accounting for clustering of girls within villages using mixed-effects regression models. Unadjusted analyses of outcomes controlled for village strata and cluster at baseline. Adjusted analyses controlled additionally for baseline levels of dropout, marriage and learning environment at school (in the form of baseline cluster means), and village type (feeder vs main), caste, and literacy status of the household head at endline. All covariates were specified *a priori*. We also planned *a priori* to conduct subgroup analyses by district.

## RESULTS

### Study population

Of the total 2457 SC/ST girls enrolled in the trial, 2275 (92.6%) were interviewed at baseline (cohort 1 = 1084; cohort 2 = 1191) and 1788 (72.8%) were interviewed at endline (cohort 1 = 872; cohort 2 = 916). In terms of trial arms, at baseline, 1111 girls (of targeted 1192) were interviewed in intervention arm whereas 1164 (of targeted 1295) in control. A total 182 girls could not be interviewed as the baseline as 60 were not traceable from the original line list of girls, 54 were migrated, 20 were married and 48 were either not available for survey, refused or did not have parental consent (**Figure 1**). Of the total 1788 girls interviewed at endline, 876 belonged to intervention whereas 912 to control. The reasons for attrition of 669 girls (316 intervention; 353 control) includes not traceable/available (41%), migrated (19%), married (30%), and others (10%). The reasons for the attrition did not vary across the intervention and control arm.

Girls were evenly distributed between cohort 1 (48%) and cohort 2 (52%), with slightly more girls living in Bagalkote (55%) compared with Vijayapura (45%) districts. Most girls (71%) lived in a “main” village, with the remainder living in “feeder” villages (**Table 1**). Socio-demographic characteristics of adolescent girls and school level characteristics were evenly distributed across trial arms at baseline (**Table 1**). The median age of girls across the arms was 13 years (IQR = 12-16), and two thirds (67%) had reached menarche. Most were from the scheduled caste (79%) with the remainder from scheduled tribes. Levels of orphanhood were high, with 17% orphaned by one or both parents. A minority (5%) were from *Devadasi* families.

**Table 1:** Assessing cluster level imbalance in characteristics of girls and trial outcomes across intervention and control arms at the beginning of the trial*

	All village clusters	Control	Intervention	Mean difference
**Individual-level summary**				
**N**	**2275**	**1111**	**1164**	
**A. Socio-demographic characteristics:**				
Cohort (1 vs 2)	1191 (52.4%)	566 (50.9%)	625 (53.7%)	-2.80
District (Bagalkote vs Vijayapura)	1016 (44.7%)	491 (44.2%)	525 (45.1%)	-0.90
Village Type (Feeder vs Main)	1608 (70.7%)	765 (68.9%)	843 (72.4%)	-3.50
Median age of adolescent girls in years (IQR)	13.0 (12-16)	13.0 (12-15)	13.0 (12-16)	0.00
Caste (ST/SC)	1788 (78.6%)	902 (81.2%)	886 (76.1%)	5.10
Menarche (N/Y)	1523 (67.3%)	751 (68.0%)	772 (66.6%)	1.40
Orphan hood (No/One or Both Parents)	379 (16.7%)	181 (16.3%)	198 (17.0%)	-0.70
Belonged to *Devadasi* family (N/Y)	112 (4.9%)	47 (4.3%)	65 (5.6%)	-1.3
Household wealth quintile (Richest/Poorest)	455 (20.0%)	227 (20.4%)	228 (19.6%)	0.80
Non-literate Household Head (Literate/Illiterate)	1415 (62.7%)	667 (60.3%)	748 (64.9%)	-4.60
**Cluster level summary (mean of means)†**				
**Number of clusters‡**	**80**	**40**	**40**	**-**
**Mean number of girls per cluster**	**28**	**28**	**29**	
**B. Sibling and school level characteristics:**				
Recent eve teasing (N/Y)	8.9 (7.7)	9.1 (9.2)	8.6 (5.9)	0.50
Girls having a sister who dropped out of school before age 16 (N/Y)	9.7 (8.6)	10.2 (9.8)	9.3 (7.2)	0.90
Girls having a sister who married <18 years (N/Y)	6.7 (7.1)	6.9 (8.1)	6.6 (6.0)	0.30
Girls who report poor learning environment in current / last school (N/Y)	7.6 (7.2)	6.2 (5.8)	9.1 (8.3)	-2.90
Girls who report poor school infrastructure in current / last school (N/Y)	12.7 (9.3)	12.5 (9.3)	12.9 (9.5)	-0.40
Girls who report harassing/bullying environment in current / last school (N/Y)	7.9 (7.2)	8.8 (7.3)	7.1 (7.0)	1.70
**C. Schooling, marriage and sexual debut at the trial start:**				
Proportion of girls who were not in school	9.4 (8.4)	8.5 (9.1)	10.2 (7.8)	-1.70
Proportion of girls who are married	6.5 (5.7)	5.8 (6.3)	7.2 (5.6)	-1.40
Proportion of girls who have sexual debut	0.8 (1.7)	0.6 (1.5)	1.0 (1.9)	-0.40
Proportion of girls married and cohabiting with husband	0.7 (1.7)	0.4 (1.3)	0.9 (1.9)	-0.50

SC/ST – scheduled-caste/scheduled-tribe, Y/N – yes/no, IRQ – interquartile range

*All statistics are n (%) unless otherwise specified.

†Mean of cluster level means of proportions (SD) of sibling and school level characteristics and outcomes at the start of the trial.

‡Control arm: 39 cluster in BL cohort-2.

Across the arms, 20% of girls were from households with the poorest wealth quintile and almost two thirds (63%) had an illiterate household head. Reports of eve teasing, sibling characteristics related to marriage and drop-out, and girl’s perceptions of their school environment were similar across trial arms (**Table 1**). Where measured, levels of primary and secondary outcomes were also similar across trial arms (**Table 1**); 9% of girls had dropped out of school at the baseline survey, 7% were married, <1% reported sexual debut and <1% were married *and* co-habiting with their husband.

### Cluster-level summaries of trial outcomes

In the end-line cross-sectional survey, the unadjusted-cluster-level summaries were similar across the trial arms for all primary and secondary outcomes (**Table 2**). The intra-cluster correlation was 1.0 for all outcomes indicating that clusters within in the invention and control areas had almost similar primary and secondary outcomes.

**Table 2:** Cluster-level summaries of the outcomes at endline*

Outcomes	Control (N = 40)	Intervention (N = 40)	Intra-cluster correlation	Risk difference (95% CI)
**Primary outcomes:**				
Proportion of girls who complete secondary school [sit 10^th^ standard exam]	74.1 (16.4)	73.3 (16.4)	1.0	0.8 (-6.5, 8.0)
Proportion of girls who are married [by trial end line]	8.4 (8.7)	11.1 (8.7)	1.0	-2.7 (-6.6, 1.1)
**Secondary outcomes:**				
Proportion of girls who start secondary school [enter into 8^th^ standard]†	91.3 (10.2)	92.8 (10.1)	1.0	-1.5 (-6.0, 2.9)
Proportion of girls who pass secondary school final year exams [pass 10^th^ standard exam]	59.7 (17.7)	56.6 (17.7)	1.0	3.1 (-4.9,11.0)
Proportion of girls who have sexual debut [by trial end line]	5.9 (6.5)	7.1 (6.5)	1.0	-1.2 (-4.0, 1.7)
Proportion of girls married and cohabiting with husband [by trial end line]	4.8 (6.3)	5.3 (6.3)	1.0	-0.5 (-3.2, 2.3)

*All statistics are mean of cluster level means of proportion (SD) unless otherwise specified.

†Control arm: 39 cluster in BL cohort-2.

### Individual-level analysis of trial outcomes

When we examined individual-level data at end-line, in both unadjusted and adjusted analyses, there was no evidence of a significant difference in the proportion of girls reporting any of the primary or secondary outcomes, by trial arm (**Table 3**). The proportion of girls completing secondary school education was 75.1% in the control arm vs 74.6% in the intervention arm and the proportion reporting marriage at end-line was 9.6% in the control arm and 10.1% in the intervention arm. Likewise, the proportion of girls who transitioned into secondary school was high in both arms (91.2% control; 92.8% intervention), and more than half of study participants passed their end of secondary school exams (60.5% control; 56.8% intervention). The proportion of girls who reported sexual debut at the follow-up survey (6.1% control; 7.0% intervention) and the proportion who reported being married *and* cohabiting with their husband (5.3% control; 4.7% intervention) was low, and similar between trial arms.

**Table 3:** Effects of the intervention on outcomes at endline

Outcomes	Summary statistics		Intervention effect*	
**Control (876 girls)**	**Intervention (912 girls)**	**Basic model**	**Adjusted model**
**Primary outcomes:**				
Proportion of girls who complete secondary school [sit 10^th^ standard exam]	658 (75.1%)	680 (74.6%)	0.99 (0.7 to 0.41), *P* = 0.987	1.01 (0.7 to 1.38), *P* = 0.961
Proportion of girls who are married [by trial end line]	84 (9.6%)	92 (10.1%)	1.09 (0.7 to 1.56), *P* = 0.658	1.00 (0.7 to 1.41), *P* = 0.978
**Secondary outcomes:**				
Proportion of girls who start secondary school [enter into 8^th^ standard] †,‡	516 (91.2%)	580 (92.8)%	1.26 (0.7 to 2.20), *P* = 0.414	1.32 (0.7 to 2.31), *P* = 0.331
Proportion of girls who pass secondary school final year exams [pass 10^th^ standard exam]	530 (60.5%)	518 (56.8%)	0.90 (0.6 to 1.22), *P* = 0.504	0.83 (0.6 to 1.15), *P* = 0.264
Proportion of girls who have sexual debut [by trial end line]	53 (6.1%)	64 (7.0%)	1.17 (0.8 to 1.71), *P* = 0.411	1.05 (0.7 to 1.55), *P* = 0.793
Proportion of girls married and co-habiting with husband [by trial end line]	46 (5.3%)	43 (4.7%)	0.92 (0.5 to 1.55), *P* = 0.766	0.83 (0.5 to 1.34), *P* = 0.447

*Odds ratio (95% CI), *P*-value for all the outcomes.

†N in summary statistics for Intervention = 625, Control = 566.

‡N in basic and adjusted model = 1191 as the data corresponding to the Cohort-2 girls at the baseline. The basic models for all the outcomes are only adjusted for village strata and cluster without any other adjustments for baseline. Adjusted models controlled for village type, caste, household literacy status, and stratum using individual-level data at the endline. Adjusted models additionally controlled for poor learning environment at school, school dropout, and marriage at baseline (in the form of cluster-level summaries).

### Sub-group analyses

When we stratified by district, in adjusted analyses, there was evidence that the Samata programme increased the proportion of girls who completed secondary school (control 73% vs intervention 77%; adjusted odds-ratio AOR = 1.54, 95% CI = 1.02-2.34) and the proportion of girls who entered into secondary school (control 90% vs intervention 96%; AOR = 3.58, 95% CI = 1.36-9.44) in Vijayapura district (**Table 4**). There was no evidence of an intervention effect in Bagalkote district. When we stratified by cohort, there was no evidence of any intervention effect on the primary and secondary outcomes (data not shown).

**Table 4:** Effects of the intervention on outcomes at endline by district

Outcomes	Bagalkote				Vijayapura			
**Summary statistics**		Intervention effect*		**Summary statistics**		Intervention effect*	
**Control (516 girls)**	**Intervention (519 girls)**	**Basic model**	**Adjusted model**	**Control (360 girls)**	**Intervention (393 girls)**	**Basic Model**	**Adjusted model**
**Primary outcomes:**								
Proportion of girls who complete secondary school [sit 10^th^ standard exam]	397 (76.9%)	379 (73.0%)	0.75	0.73	261 (72.5%)	301 (76.6%)	1.43	1.54
			(0.4 to 1.19)	(0.4 to 1.14)			(0.8 to 2.33)	(1.0 to 2.34)
			0.226	0.169			0.155	0.042
Proportion of girls who are married [by trial end line]	56 (10.9%)	62 (11.9%)	1.18	1.12	28 (7.8%)	30 (7.6%)	0.97	0.79
			(0.7 to 1.82)	(0.7 to 1.69)			(0.5 to 1.69)	(0.4 to 1.38)
			0.467	0.606			0.921	0.406
**Secondary outcomes:**								
Proportion of girls who start secondary school [enter into 8^th^ standard]†,‡^@^	290 (92.1%)	330 (90.2%)	0.73	0.90	226 (90.0%)	253 (96.3%)	3.27	3.58
			(0.3 to 1.43)	(0.4 to 1.72)			(1.2 to 8.40)	(1.3 to 9.44)
			0.356	0.753			0.014	0.010
Proportion of girls who pass secondary school final year exams [pass 10^th^ standard exam]	309 (59.9%)	290 (55.9%)	0.86	0.78	221 (61.4%)	228 (58.0%)	0.98	0.92
			(0.5 to 1.26)	(0.4 to 1.27)			(0.6 to 1.57)	(0.6 to 1.42)
			0.432	0.317			0.941	0.709
Proportion of girls who have sexual debut [by trial end line]	30 (5.8%)	34 (6.6%)	1.15	1.06	23 (6.4%)	30 (7.6%)	1.18	1.05
			(0.6 to 1.91)	(0.6 to 1.82)			(0.6 to 2.08)	(0.5 to 1.89)
			0.587	0.819			0.559	0.869
Proportion of girls married and co-habiting with husband [by trial end line]	28 (5.4%)	25 (4.8%)	0.92	0.93	18 (5.0%)	18 (4.6%)	0.97	0.75
			(0.4 to 1.71)	(0.5 to 1.65)			(0.3 to 2.39)	(0.3 to 1.79)
			0.786	0.793			0.949	0.514

*Odds ratio (95% CI), *P*-value for all the outcomes.

†N in summary statistics for Bagalkote (Intervention = 366, Control = 315) and Vijayapura (Intervention = 259, Control = 251).

‡N in basic and adjusted model = 1191 as the data correspond to the Cohort-2 girls at the baseline. The basic model for all the outcomes are just adjusted for village strata and cluster without any other adjustments for baseline. Adjusted models controlled for village type, caste, household literacy status, and stratum using individual-level data at the endline. Adjusted models additionally controlled for poor learning environment at school, school dropout, and marriage at baseline (in the form of cluster-level summaries).

### Sensitivity and attrition analyses

We conducted sensitivity analyses, which included marriage and schooling data from 544 girls who did not participate in the end-line survey, but for whom a family member provided data on the outcomes (266 control; 278 intervention). When we included data from these girls in the individual level analyses, results were similar to the main findings (Appendix Table 1 in **Online Supplementary Document**). In addition to the sensitivity analyses, an attrition analysis was done to understand whether the loss to follow-up of the girls from baseline to endline was random or it has any bearing on the outcomes due to loss-to-follow-up (LFU) of girls with specific characteristics at the endline. Two scenarios were envisaged. First, all the loss to follow-up girls in the intervention and control areas completed the secondary education and no one got married and second, vice-a-versa. The attrition analysis did not find any significant difference in the outcomes across the two arms and suggested that LFU of girls from baseline to endline was random. For example, the rates of secondary school completion were 69.7% vs 66.9% and rates of child marriages were 7.4% vs 7.7%, respectively, in control and invention if we assumed that all the missing girls in the control and intervention completed secondary school and no one got married. Similarly, the rates of secondary school completion and marriage were 46.4%vs 43.5% and 30.6% vs 31.1%, respectively in the control and intervention if all missing girls did not complete secondary school and everyone got married by endline. In addition to above, if we consider an extreme scenario where all the girls from intervention completed school and no one got married whereas just an opposite situation in control, we found significantly lower rates of secondary school completion (46.4% vs 66.9%; *P* < 0.05) and higher rates of early marriage (30.6% vs 7.7%; *P* < 0.05) in control and intervention, respectively. This imply that results are sensitive to the missing cases only in extreme cases.

### Associations between school drop-out and child marriage

We examined associations between secondary school continuation and completion with child marriage at endline. Overall, the proportion of girls who were married was higher among girls who did not sit in the secondary school exam compared to those who did (24.5% vs 4.8%, respectively) and those who did not pass 10^th^ standard exam with those who passed (16.8% vs 4.9%). There was no significant difference in marriage rates among girls who sat in the exam by arm. However, there was weak evidence of lower likelihood of marriage among girls in the intervention arm who passed the exam compared to girls in the control arm (AOR = 0.53, 95% CI = 0.26-1.07). When we stratified by district, girls in the intervention in Vijayapura district who completed or passed the exam were significantly less likely to be married compared with girls in the control area (control 4.6% vs intervention 1.9%, AOR = 0.22, 95% CI = 0.07-0.76; control 4.9% vs intervention 0.9%, AOR = 0.04, 95% CI = 0.01-0.39, respectively). There were no significant differences in marriage rates among girls out of school in Vijayapura district between control and intervention arms.

## DISCUSSION

In this study, we found no significant differences between trial arms, in our primary and secondary outcomes. Thus, we found no significant difference in the proportion of girls who entered into secondary school (standard 8), who completed secondary school (standard 10), or who passed the end of secondary school exam (standard 10 final exam). We also found no significant difference in the proportion of girls who were married (age 15/16 years), married and cohabiting with their husband, or reported sexual debut at endline. However, when we stratified by district, in Vijayapura, we observed a significant increase in the proportion of girls who enrolled into and completed secondary school in the intervention villages, compared with the control villages.

### Secular trends and government programmes

In our study, the proportion of girls experiencing the trial outcomes (school drop-out/marriage) at end-line in both intervention and control arms was far lower than we had anticipated based on evidence from the district and state level data at the time of designing the intervention. A time-trend analysis of the District Level Household Survey and National Family Health Survey data for 2002-2015 found large secular changes in child marriage and secondary school completion among girls across the state (manuscript submitted to Lancet Global Health). These changes were not only observed at the state-wide population level but also in rural SC/ST communities. Thus, the rates of child marriage among rural SC/ST women aged 18-24 years have fallen from 72.8% in 2002 to 24.7% in 2015, and rates of secondary school completion among rural SC/ST girls aged 15-17 have increased from 13.9% in 2002 to 47.9% in 2015 at the state level. Improvements in secondary school completion and levels of child marriage were seen in the two districts of our study, as well as in other States in India [8]. These changes demonstrate substantial success at the national and state level to improve education for girls, and are hugely important with likely positive inter-generational benefits for girls’, and their children.

We also examined if any other programmes may have impacted on our trial findings, and found a number of national, state and district-wide government-led initiatives that were implemented between 2011 and 2017 in our study districts (Figure S1 in **Online Supplementary Document**). These programmes aimed to provide financial support, additional tutorial classes and ensure schools were safe, to encourage school completion and grade attainment, and are similar to components of the Samata intervention. The impact was reflected by high levels of exposure to scholarships, ‘mission 100^’^ exam-preparation classes for poor performing students, livelihood training and tutorial classes by girls in our study regardless of trial arm (Table S2 in **Online Supplementary Document**). Thus, substantial changes in school completion and child marriage were observed due to government programmes that were consistent with those implemented by the Samata programme during the study.

It is notable that despite this backdrop of secular changes and government-led programmes, the Samata programme was still able to achieve a modest impact on schooling outcomes among SC/ST girls in one district. This suggests that additional gains can still be made through intensive programming and/or that families in Vijayapura district were readier for change through the cumulative impact of previous programmes in this district.

### Intervention design

Our programme design differed from previous secondary school and child marriage trials. Whereas most trials have used economic components as the main intervention, the Samata programme worked with multiple stakeholders (girls, boys, families, school teachers, community leaders) to change structural factors and gendered norms around early marriage and the value of girl’s education. In our intervention, we linked girls and families in the intervention villages to government-funded economic initiatives designed to promote girl’s education, but these funding schemes were also available in the control areas and were accessed by families in both arms (Table S2 in **Online Supplementary Document**).

Our key hypothesis was that child marriage and school drop-out are linked; and that preventing the latter will delay the former. However, our sub-analyses in this paper and our previous qualitative findings suggest that while this is sometimes true, unmarried girls drop out of school for other economic, social and academic reasons and some married girls remain in secondary school despite early marriage [38]. From a programmatic perspective, similar structural forces serve to determine both outcomes, but it’s worth noting that in this context, increasing secondary school entry and completion among girls in Vijayapura district was not associated with concomitant changes in child marriage.

It may be that stronger social and cultural forces affect family decision-making on marriage than on education, and these entrenched views are made earlier in a girls’ life and are more challenging to change. Evidence from the global literature from sub-Saharan Africa and South America suggests that providing financial incentives is effective in keeping girls unmarried [20,24-26,29] and in secondary school [20,21,24-27,29,32,34], and thus may be sufficient to overcome the economic and cultural forces that determine a girls marital, and consequent educational, outcomes in impoverished settings. However, this raises questions over the sustainability of such approaches. In addition, the contexts in sub-Saharan Africa and South America differ substantially to south Asia; whereas reducing high HIV-incidence among adolescent girls has been a key focus of the trials in sub-Saharan Africa, in south Asia where HIV rates are low, secondary school drop-out and child marriage are high and strongly linked with gender inequitable norms. Recent trials in India and Bangladesh provide the first evidence that providing decent livelihood options for girls or providing financial incentives can significantly impact on secondary school retention and child marriage rates in south Asian contexts too [26,29].

### Programme implementation

We were interested to understand why we found an impact of *Samata* on schooling outcomes in Vijayapura but not in Bagalkote district. While outreach workers delivering the intervention were of similar ages in both districts, those in Vijayapura had higher educational qualifications and worked on the intervention longer than those in Bagalkote (Table S3 in **Online Supplementary Document**). In addition, the programme was delivered more intensively (frequency of contacts) in Vijayapura district (Table S3 in **Online Supplementary Document**).

The qualitative data provides important insights in terms of programme implementation and reveals the pivotal role that families play in decision making about marriage and education for their daughters [38]. The qualitative data also suggests that the primary norm discouraging school attendance may not be undervaluing girl’s education as anticipated, but concern about reputational risk to family honour. The initial focus of our intervention was empowering adolescent girls and working with boys to make them more respectful and supportive of their sisters. Following a mid-line programme review (2016), we re-focused our efforts on engaging with parents/carers, but this was likely too late in the study to have had an impact. Based on our experience, we would recommend future programmes focus more intensely on working with family members in the first instance and start when girls are younger (pre-menarche).

One of the main trial aims was to delay sexual debut and to delay entry into sex work (through keeping girls in school), due to previously reported high rates of HIV among *Devadasi* sex workers. However, our surveys found far smaller numbers of girls reporting to be *Devadasi* than we anticipated. This could be the result of under-reporting due to social desirability bias (sex work is illegal in India) or it could be that SC/ST girls from *Devadasi* families had dropped out of school before year 7 and were therefore not included in our study sample. Similarly, by enrolling girls in the final year of primary school we may have already missed those most at risk of school drop-out and child marriage. Future interventions may need to focus on younger girls (eg, aged 9/10 years) and their families, and target those at highest-risk. Indeed, some researchers suggest programmes should invest in the poorest girls in the poorest communities a year or two before puberty to make a difference [39].

### Strengths and limitations

Our results should be generalizable to SC/ST girls in India. We included all SC/ST girls enrolled in 7^th^ standard in our sample, had a large number of clusters, 100% of selected villages agreed to participate and no villages dropped out of the study. Analysis of baseline balance suggested the randomisation process worked well and the study design was robust. Our trial outcomes were reliant on self-reported measures and were likely to be subject to reporting bias. As there has been a lot of publicity around the illegality of child marriage, child marriage is likely to be under-reported and biased in the same direction in both trial arms. Likewise, studies from LMIC globally suggest that parents and their children are often aware of the importance of education [14], and reports on school drop-out were likely to be biased in the opposite direction in both arms. As noted above, the prevalence of our trial outcomes differed substantially from what we had estimated at trial start, due to large secular changes in child marriage and girl’s secondary education which have been taking place across India. As such, we do not think that increasing the sample size would have changed the trial findings. Our loss-to-follow-up was higher than anticipated with ~ 27% of the study population not available for interview at end-line. Even though it is likely that girls who were not available for interview were more likely to have been married or dropped-out of school than those available for interview, we do not think this affected the overall trial results as loss-to-follow-up was similar between trial arms and sensitivity analyses using data from 544 ‘missing girls’ found the study findings remained the same.

A cluster-RCT may not be the most appropriate evaluation design to measure the impact of programmes which take a normative approach, as control arms may not remain “pure” and norm change happens in stages – first among those most ready for change, and later among those less ready for change who may only change during subsequent programmes. Thus, a trial may capture those girls and families who are most ready for change but miss those who were brought to a higher level of readiness for change.

## CONCLUSIONS

This is the first trial to take a multi-level approach to address normative and structural factors hypothesised to encourage child marriage and school drop-out among adolescent girls. The Samata intervention was associated with improvements in secondary school enrolment and completion among SC/ST adolescent girls in one district but did not impact on child marriage outcomes. Low school drop-out and marriage rates at end-line, coupled with exposure to various government-led campaigns in the control villages, makes it difficult to assess the value of this multi-level, norms-based approach. Additional research is under way to understand the impact of the Samata intervention on factors hypothesised to be on the causal pathway for the trial primary outcomes and to further explore the role of various social norms on influencing girl’s education and marriage. Our findings highlight the need for further research to identify interventions which target the sizeable minority of SC/ST adolescent girls who remain at risk of secondary school drop-out and child marriage.

## Additional material

Online Supplementary Document
