# Supplementary material for: The Samata intervention to increase secondary school completion and reduce child marriage among adolescent girls: results from a cluster-randomised control trial in India
Source: J Glob Health. 2019 Jun 25;9(1):010430. doi: 10.7189/jogh.09.010430 (PMC6684866; doi:10.7189/jogh.09.010430)
Supplement: Online Supplementary Document [file jogh-09-010430-s001.zip › 6_Appendix Tables.docx]

**Table S1.** Sensitivity analysis depicting selected schooling and marriage outcomes by Trial arm at endline

|  | **Overall** | |  | **Bagalkote** | |  | **Vijayapura** | |
| --- | --- | --- | --- | --- | --- | --- | --- | --- |
|  | **Control** | **Intervention** |  | **Control** | **Intervention** |  | **Control** | **Intervention** |
| **Number of girls selected for cross-sectional survey** | 1192 | 1265 |  | 651 | 673 |  | 541 | 592 |
| Number of girls completed face-to-face endline interview | 876 | 912 |  | 516 | 519 |  | 360 | 393 |
| Number of girls did not participated in face-to-face interview but outcome information collected indirectly* | 266 | 278 |  | 117 | 133 |  | 149 | 145 |
| Number of girls whose outcome information could not be collected | 50 | 75 |  | 18 | 21 |  | 32 | 54 |
| **Number of girls included in sensitivity analysis** | 1142 | 1190 |  | 633 | 652 |  | 509 | 538 |
| **Outcomes** |  |  |  |  |  |  |  |  |
| Proportion of girls who pass secondary school final year exams[pass 10^th^ standard exam] | 581 (50·9%) | 566 (47·6%) |  | 329 (52·0%) | 302 (46·3%) |  | 252 (49·5%) | 264 (49·6%) |
| Proportion of girls who are married [by Trial end line] | 179 (15·7%) | 180 (15·1%) |  | 98 (15·5%) | 114 (17·5%) |  | 81 (15·9%) | 66 (12·3%) |
| All statistics are n (%) unless otherwise specified. *Information on girl’s schooling and marriage was collected from the parents, family member or neighbours. | | | | | | | | |

**Table S2:** Exposure of girls to different schemes, tutorial classes, and life skill programmes by Trial arm at endline

|  | **Control**  **(n=876)** |  | **Intervention**  **(n=912)** |
| --- | --- | --- | --- |
| **A. Financial assistance** |  |  |  |
| Incentive scholarship | 405 (46·2%) |  | 405 (44·4%) |
| Scholarship for 10^th^ standard school going girls | 479 (54·7%) |  | 527 (57·8%) |
| Pre-metric scholarship | 450 (51·4%) |  | 459 (50·3%) |
|  |  |  |  |
| **B. Educational assistance** |  |  |  |
| Tutorial classes | 397 (45·3%) |  | 487 (53·4%) |
| Mission-100^*^ | 65 (14·9%) |  | 68 (14·2%) |
|  |  |  |  |
| **C. Life skill programme** |  |  |  |
| Career counselling | 314 (35·9%) |  | 438 (48·0%) |
| Livelihood training | 255 (29·1%) |  | 295 (32·3%) |
| Sabala programme | 68 (7·8%) |  | 144 (15·8%) |
| All statistics are n (%) unless otherwise specified. ^*^Applicable only for Cohort-2 girls (Control=436; Intervention=480). | | | |

**Table S3.** Profile of the ORWs and intensity of programme exposure among adolescent girls in intervention area by district at endline

|  | **Overall** |  | **Bagalkote** |  | **Vijayapura** |
| --- | --- | --- | --- | --- | --- |
| **A. Staff detail** |  |  |  |  |  |
| **Number of outreach workers (ORW) recruited by the intervention** | **38** |  | **21** |  | **17** |
| Age (years), mean (SE) | 33·5 (1·6) |  | 34·3 (1·1) |  | 32·5 (3·3) |
| Years of schooling, mean (SE) | 13·8 (0·4) |  | 12·5 (0·6) |  | 15·4 (0·3) |
| Duration of work in Samata (months), mean (SE) | 29·2 (3·3) |  | 23·7 (3·9) |  | 36·0 (5·3) |
| Percentage of ORWs worked more than the average duration of work within the district | 18 (47·4%) |  | 9 (42·9%) |  | 9 (52·9%) |
|  |  |  |  |  |  |
| **B. Intensity of programme exposure among adolescent girls** |  |  |  |  |  |
| **Number of girls participated in the endline survey*** | **912** |  | **519** |  | **393** |
| Frequency of contact by ORW |  |  |  |  |  |
| Not contacted | 18 (1·9%) |  | 13 (2·5%) |  | 5 (1·3%) |
| At least once a week | 343 (36·6%) |  | 179 (34·5%) |  | 164 (41·7%) |
| At least once a month | 236 (25·9%) |  | 124 (23·9%) |  | 112 (28·5%) |
| At least once in a quarter | 315 (34·5%) |  | 203 (39·1%) |  | 112 (28·5%) |
| All statistics are n (%) unless otherwise specified. *Only from the intervention area | | | |  |  |
|  | | | | | |
