# Supplementary material for: The Samata intervention to increase secondary school completion and reduce child marriage among adolescent girls: results from a cluster-randomised control trial in India
Source: J Glob Health. 2019 Jun 25;9(1):010430. doi: 10.7189/jogh.09.010430 (PMC6684866; doi:10.7189/jogh.09.010430)
Supplement: Online Supplementary Document [file jogh-09-010430-s001.zip › 5_Appendix S1 Intervention design.pdf]

STRIVE

Tackling the structural drivers of HIV

Samata: Keeping girls in secondary school

Project Implementation Design

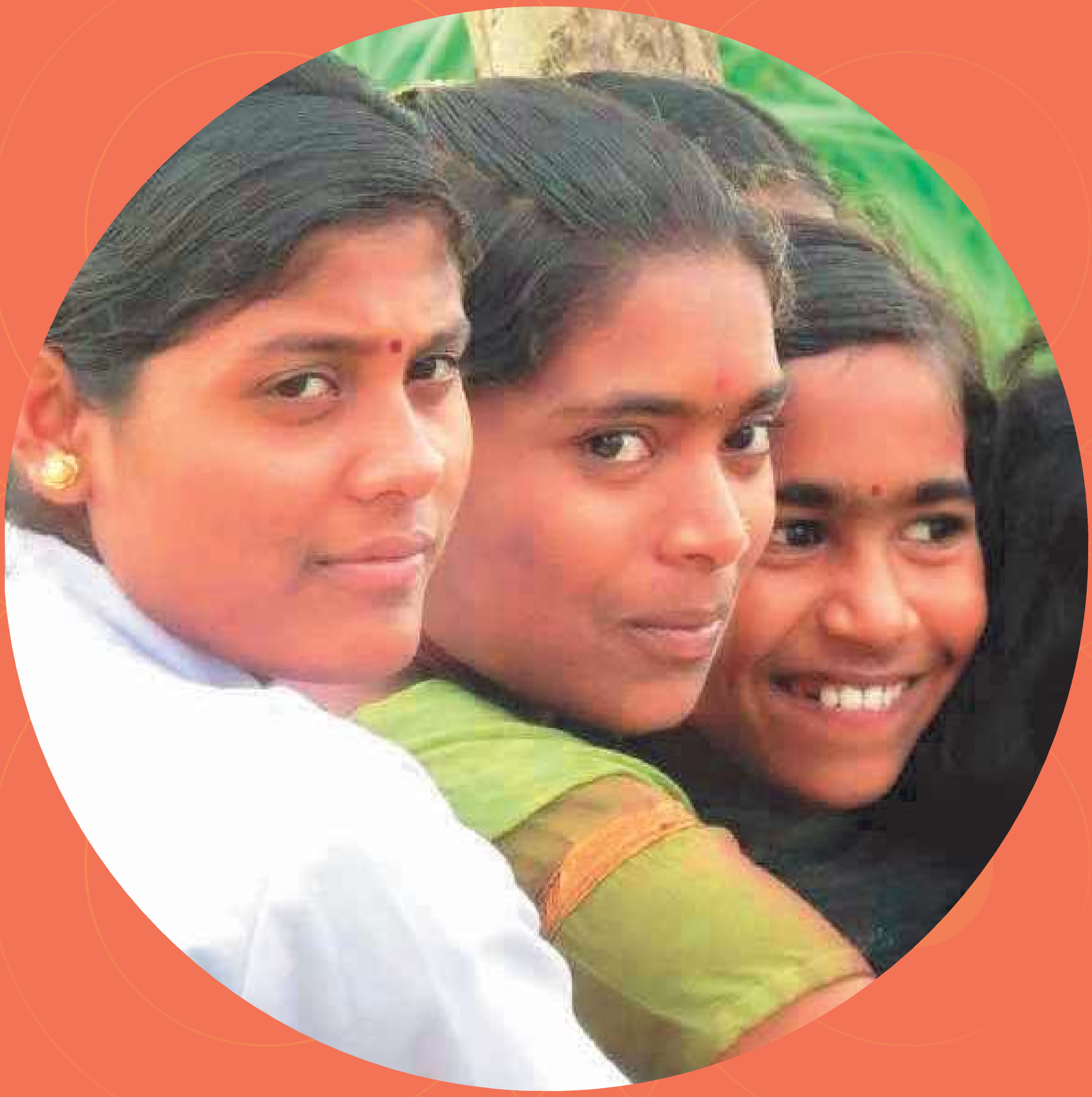

KHPT Karnataka Health Promotion Trust

London School of Hygiene & Tropical Medicine

UKaid from the Department for International Development

Bill & Melinda Gates foundation

University of Manitoba

World Bank

**Authors**	:	T. Raghavendra and Brooks Anderson
**Editor**	:	Brooks Anderson
**Cover photo**	:	Yellamma Peerappa Chalvadi, IXth grade, Government High School Tangadagi, Bijapur
**Photos**	:	Priya Pillai
**Design**	:	M.B.Suresh Kumar, Artwist Design Lab
**Acknowledgements**	:	Parinita Bhattacharjee, Shajy Isac, Prakash Javalkar and Sapna Nair, Lori Heise, Tara Beattie, Annie Holmes and Priya Pillai
**Copyright**	:	© KHPT, March 2013
**Publisher**	:	Karnataka Health Promotion Trust,<br>IT/ BT Park, 5th Floor,<br># 1-4, Rajajinagar Industrial Area<br>Behind KSSIDC Administrative Office<br>Rajajinagar, Bangalore- 560 044<br>Phone: 91-80-40400200<br>Fax: 91-80-40400300<br>www.khpt.org

Samata: Keeping girls in secondary school

Project Implementation Design

Karnataka Health Promotion Trust

Project – Samata

Bijapur & Bagalkot

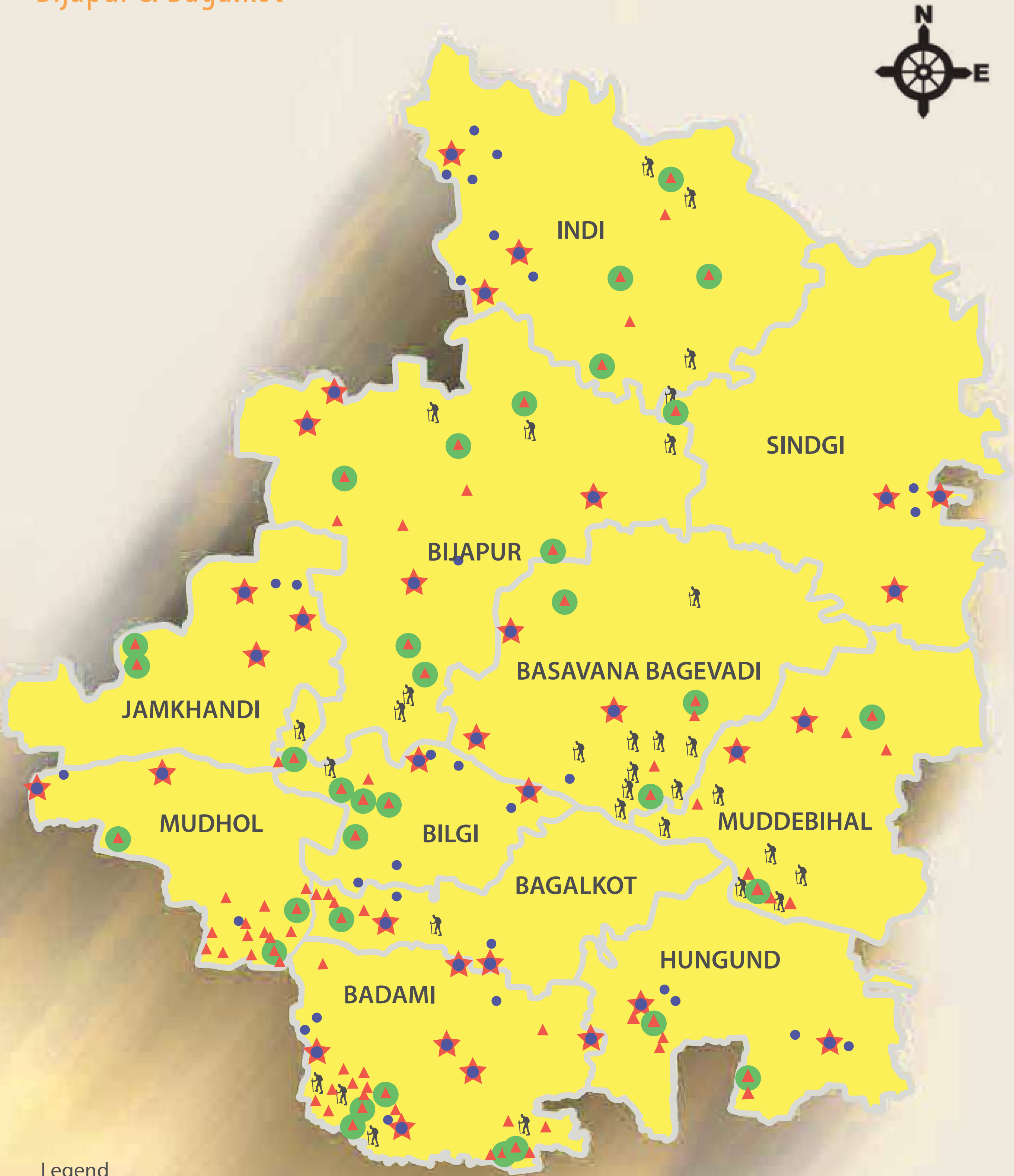

Legend

- Intervention- HS Village
- Intervention- HPS Village
- Control- HS Village
- Control- HPS Village

Samata's implementation has been supported by STRIVE, the World Bank, and the Government of Karnataka. The plan was prepared with support from STRIVE, which is funded by UK Aid from the Department for International Development (DFID). STRIVE is a research consortium based at the London School of Hygiene and Tropical Medicine, with partners in India, Tanzania, and South Africa, investigating and addressing the structural forces – in particular, stigma, gender-based violence, poverty, and drinking norms – that combine in various ways to amplify vulnerability to HIV transmission and to undermine prevention.

The views expressed herein are those of the authors and do not reflect the official policy or position of the UK Department for International Development, the World Bank, or the Government of Karnataka.

Contents

Abbreviations vi

Executive Summary viii

I. Introduction 1

The High Price of Gender Disparity in Education 2

Samata's Goal 3

Background 4

Delayed Socioeconomic Development 4

Poverty 5

Gender Norms 6

A Culture that Prizes and Privileges Men 7

Underage Marriage 8

Belonging to a Scheduled Caste or Scheduled Tribe 10

Illiteracy and Low Levels of Education 10

The Devadasi Tradition 10

Boys' Misconduct in and outside of School 11

Ill-Equipped, Understaffed Schools and Poorly Performing SDMCs 12

Problem Summary 14

2. Project Rationale 15

Why Promote Girls' Education? 16

Insights for Designing Effective Interventions to Promote Girls' Education 17

Summary: Project Rationale 20

3. Theory of Change 21

The Problem 22

Barriers 23

Interventions 24

Outputs 25

Outcomes 26

Impact 27

4. Project Implementation 28

Intervention with Schools 30

Intervention with SC/ST Girls 36

Intervention with Families of Adolescent Girls 43

Intervention with Boys 47

Intervention with Community 51

Intervention with State, District, and Block Level Officials and the Media 53

5. Management Structure 63

6. Monitoring and Evaluation 70

References 96

Abbreviations

**AG**	Adolescent Girl
**AGP**	Adolescent Girls Project
**AIDS**	Acquired Immune Deficiency Syndrome
**ANC**	Antenatal Clinic
**AY**	Academic Year
**CBO**	Community-Based Organisation
**CCL**	Centre for Child and the Law
**CSR**	Child Sex Ratio
**DLHS**	District-Level Household and Facility Survey
**DSS**	Dalit Sangarsh Samithi
**FSW**	Female Sex Worker
**GER**	Gross Enrolment Ratio
**GoI**	Government of India
**GoK**	Government of Karnataka
**HDR**	Human Development Report
**HIV**	Human Immunodeficiency Virus
**HM**	Headmaster or Headmistress
**ICHAP**	India-Canada Collaborative HIV/AIDS Project
**ICRW**	International Center for Research on Women
**IHAT**	India Health Action Trust

**KHPT**	Karnataka Health Promotion Trust
**KiA**	Knowledge into Action
**MHRD**	Ministry of Human Resource Development
**NFHS**	National Family Health Survey
**NGO**	Non-Governmental Organisation
**NRHM**	National Rural Health Mission
**OoSC**	Out of School Children
**ORW**	Outreach Worker
**PIP**	Programme Implementation Plan
**PP**	Parivartan Plus
**PRI**	Panchayat Raj Institution
**RTE**	Right to Education (Act) (also known as the Right of Children to Free and Compulsory Education Act)
**SC**	Scheduled Caste
**SDMC**	School Development and Monitoring Committee
**SSA**	Sarva Shiksha Abhiyan
**ST**	Scheduled Tribe
**STRIVE**	Structural Drivers of the HIV Epidemic
**ToT**	Training of Trainers

4 Project Implementation

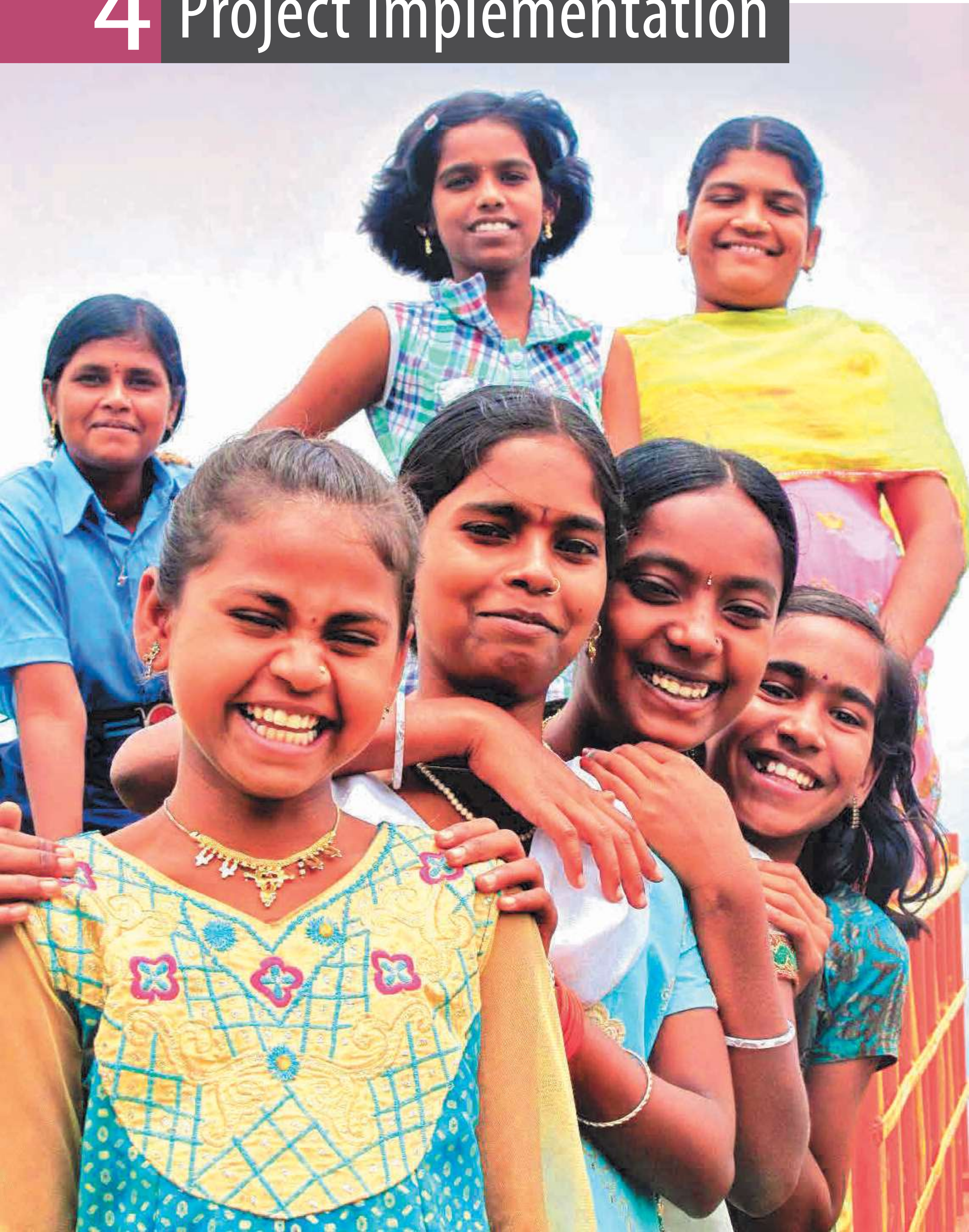

Project Samata will cover 3600 adolescent girls and 1800 families in 119 villages and 69 high schools in Bijapur and Bagalkot Districts. The project duration is for a period of five years, from July 2012 to June 2017, and is structured in three phases: i) planning and piloting (July 2012 – June 2013) , ii) implementation (July 2013 – June 2016), and iii) evaluation, consolidation, and dissemination (July 2016 – June 2017). The one-year planning phase was spent conducting assessments to inform Samata's design, and piloting intervention strategies. The three-year implementation phase, which is featured in this document, will focus on implementing the intervention. The fifth year will be for consolidation, evaluation, and dissemination.

Samata will intervene simultaneously across the social spectrum to improve adolescent girls' educational attainment.

In light of the recommendations found in several documents, Samata will intervene simultaneously across the social spectrum to increase adolescent girls' educational attainment. Samata will intervene with school staff and SDMCs, adolescent girls and their families, boys, community groups and local governing bodies, and officials from the Department of Education and the media.

Interventions with Schools, Teachers and SDMCs

Samata will work with schools and SDMCs to make girls' education more relevant, rewarding, safe, and responsive to girls' needs.

Objectives

Long-Term

- To increase accessibility and expand opportunities for girls to enter and continue formal secondary education
- To improve the capacities and accountability of schools to be responsive to girls' needs, and facilitate their entry and retention in school

Immediate

- To support schools to develop and implement action plans to encourage entry and retention of girls in secondary school
- To facilitate schools to develop and implement action plans to make schools safer for girls (anti-harassment, safe toilets, etc.)
- To improve the tracking of girls in schools by school teachers and SDMCs
- To improve positive attitudes on gender equality among teachers and SDMC
- To support schools to build leadership among girls

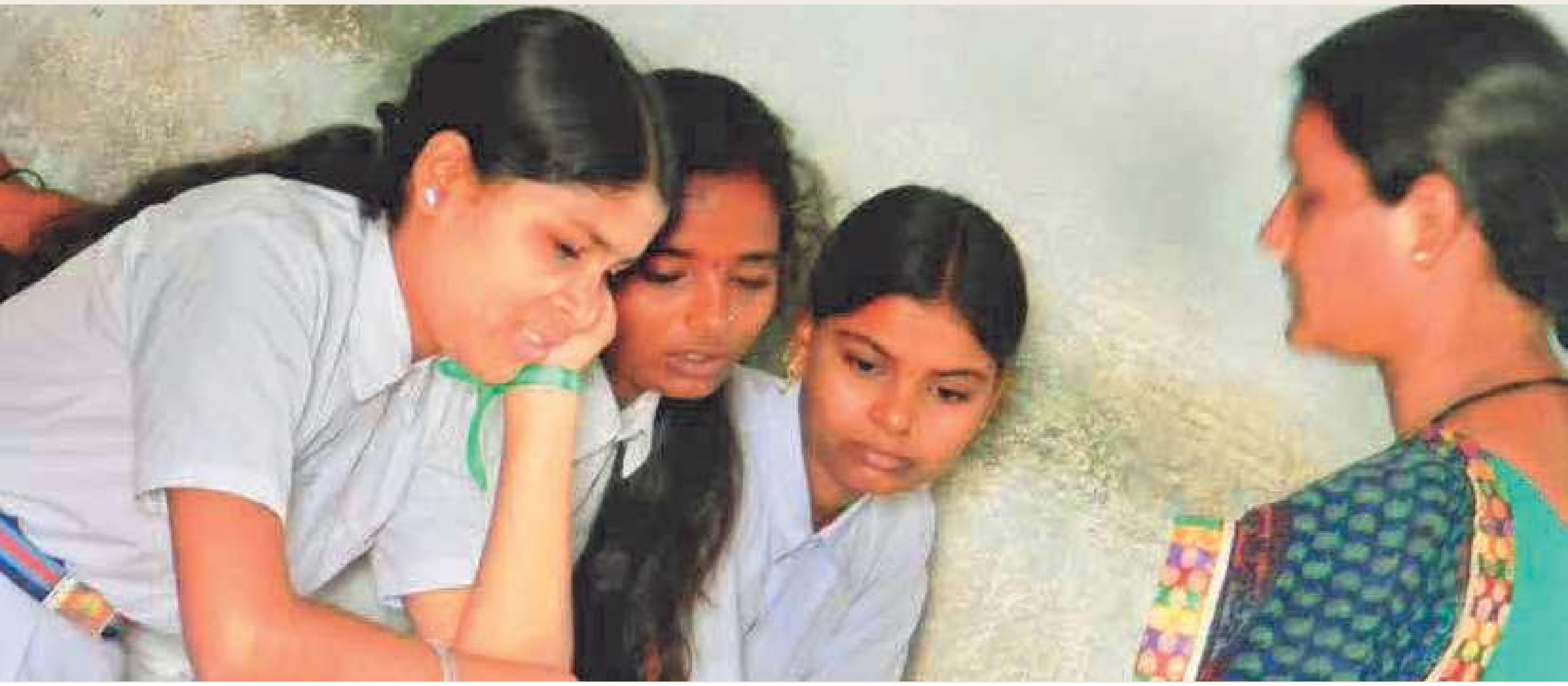

Framework of Expected Outputs, Indicators, and Means of Verification

Output – 1	Indicators	Means of Verification	Target
Skills and capacities of school staff and SDMCs built to conduct gender analysis and prepare school development plans towards girls' entry and retention in school	❖ Number of trainings conducted for teachers and number of teachers trained<br>❖ Number of trainings conducted for SDMCs and number of SDMCs members trained<br>❖ Number of schools that do gender analysis and develop an action plan to address the needs<br>❖ Number of schools that implement their action plans	❖ Teachers training reports & attendance lists<br>❖ Copies of action plans developed by schools	❖ 605 teachers from 69 schools trained<br>❖ 690 members (10 members from each SDMC from 69 SDMCs) trained<br>❖ 100% of schools (69 schools) develop and implement plans

Activities

- Assess teachers, their capacities, and trainings undergone by them so far
- Develop a two day curriculum for training the teachers and SDMCs
- Develop a team of master trainers from the education departments and regular training institutes
- Advocate for deputation of teachers for training
- Conduct gender training for teachers and SDMCs members using the curriculum
- Train SDMCs
- Develop an action plan by end of the training to initiate activities in school to promote gender equity

Output – 2	Indicators	Means of Verification	Target
Simple tools and job aids are available with school staff and SDMC for tracking entry and retention	Number of teachers trained to use the tracking tool<br>Number of schools covered in training on tracking tool<br>Number of schools using the tool for tracking	Training reports<br>Reports of usage of tool by the schools<br>Monthly progress report	605 teachers trained in tracking tool<br>69 schools covered in training<br>69 of the schools correctly using the tools to track students

Activities

- Assess existing methods of tracking students
- Develop tools for teachers to annually map and track vulnerable girls by using classes 7 and 8 enrolment lists from the area's upper primary schools, and pilot the tools in selected schools
- Advocate with schools to introduce the tool
- Train teachers to use the tool for profiling and tracking
- Monitor and support teachers to conduct gap analysis and use the tool to improve entry and retention

Output – 3	Indicators	Means of Verification	Target
Schools have policies that ensure a safe environment and participation of girls in school	- Number of workshops and meetings to conceive policies<br>- Attendance at workshops and meetings<br>- Number of schools that institute policies to promote girls' enrolment and retention in school<br>- Number of schools that institute safety measures	- Monthly progress reports<br>- Activity reports from the schools	- 69 workshops conducted<br>- 69 high schools have measures promoting girls' enrolment, retention, safety and participation

Activities

- Assist schools to institute safety measures for girls supported by a buddy system that includes peers and teachers to enable reporting and redressal of sexual harassment of girls.
- Initiate girl-friendly services in the schools like separate toilets for girls, special events for promoting girls' leadership, etc.

Output – 4	Indicators	Means of Verification	Target
Schools have leadership and career counselling programmes for adolescent girls	❖ Number of workshops for schools<br>❖ Number of schools that organised career counselling sessions and number of adolescent girls who attended<br>❖ Number of inter-school sports and cultural events conducted<br>❖ Number of leadership and personality development programmes conducted and number of adolescent girls who attended	❖ Training reports<br>❖ Event reports	❖ 69 schools have career counselling sessions (one per school)<br>❖ 3600 adolescent girls linked to schemes<br>❖ Four experiential learning events conducted yearly<br>❖ Eight inter-school sports and cultural events conducted yearly<br>❖ Four leadership and personality development trainings

Activities

- Organise career counselling sessions through schools on career options.
- Support schools in establishing links for schemes meant for adolescent girls.
- Collaborate with the school to organise intra- and inter-school sports and cultural meetings for adolescent girls that build their confidence and leadership skills, and challenge gender norms.
- Organise special leadership and personality development programmes for the adolescent girls.

Key Activities and Timelines

Key Activities	Jun-Aug, 2013	Sep-Nov, 2013	Dec-Feb, 2014	Mar-May, 2014	Jun-Aug, 2014	Sep-Nov, 2014	Dec-Feb, 2015	Mar-May, 2015	Jun-Aug, 2015	Sep-Nov, 2015	Dec-Feb, 2016	Mar-May, 2016
Assess teachers, their capacities, and trainings undergone by them so far.												
Develop a two-day curriculum to train teachers and SDMCs.												
Identify and develop a team of master trainers from the education departments and regular training institutes.												
Conduct gender training for teachers.												
Train SDMCs.												
Develop an action plan by end of the training to undertake activities in school to promote gender equity.												
Develop tools for teachers to annually map and track the vulnerable girls, and pilot the tools in selected schools.												
Train teachers to use the tool for profiling and tracking.												
Conduct gender analysis of activities in schools to understand existing practices.												
Instituting safety measures in schools for girls supported by a buddy system that includes peers and teachers to enable reporting and redressal of sexual harassment of girls.												
Collaborate with the school to organise intra- and inter-school sports and cultural meetings that build adolescent girls' confidence and leadership skills, and challenge gender norms.												
Organise special leadership and personality development programmes for the adolescent girls.												

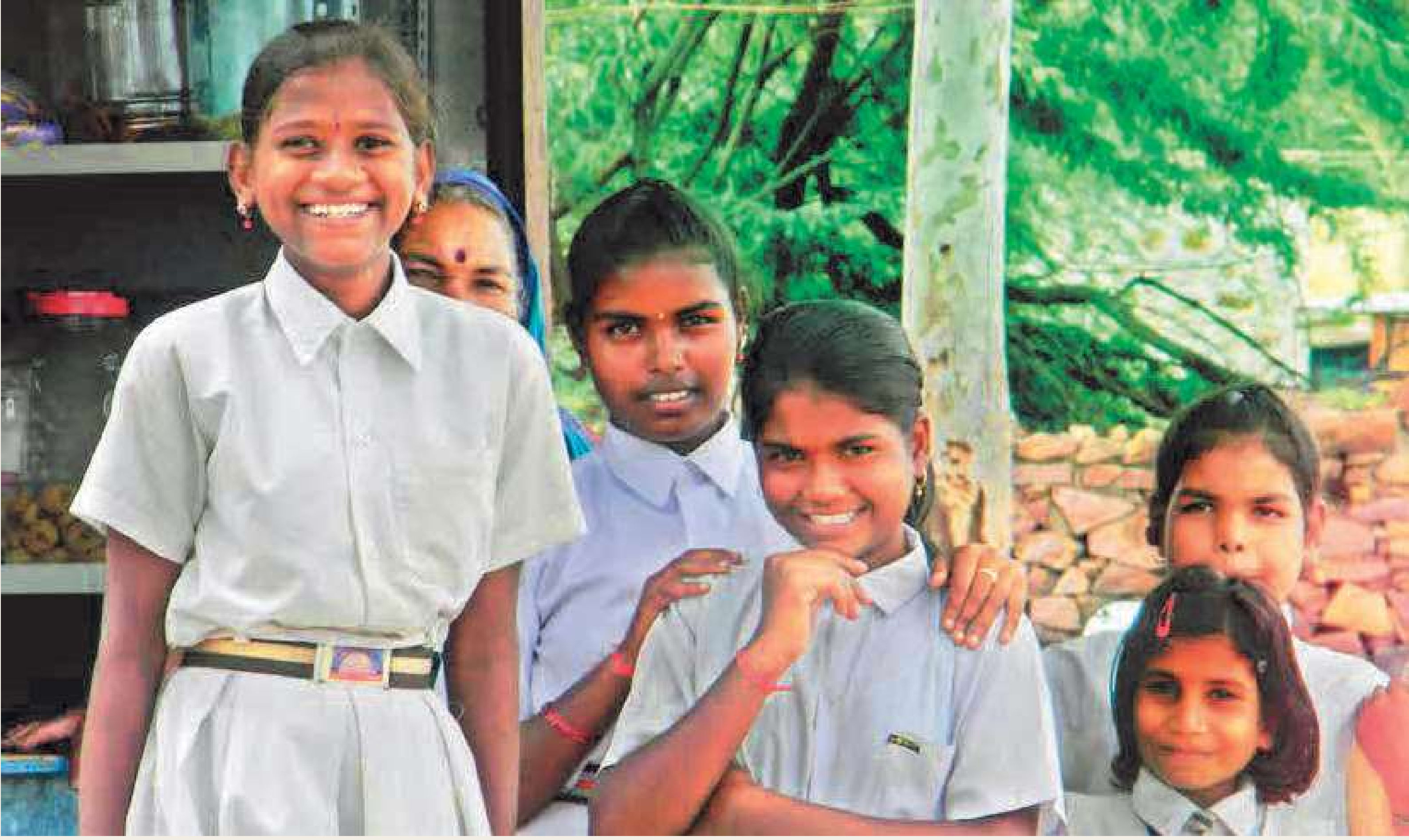

Intervention with SC/ST Girls—Classes 7 to 10 from Intervention Schools and Villages

Samata will strengthen the self-esteem and awareness of adolescent girls to enable them to make informed choices and empower them to collectively confront and overcome the issues they face. The project will encourage girls to collectively demand changes in attitudes, services, and justice systems. Organising adolescents as a strong force will help them negotiate with local governing bodies and exercise their rights. 'Champions of change' will be identified and assisted to form support groups for adolescents living in their village's vicinity. These groups will be strengthened to engage with families and others in the community and negotiate necessary changes in attitudes, behaviors, actions, and services at the community and district level.

Samata will strengthen the self-esteem of adolescent girls and empower them to collectively confront and overcome the issues they face.

Group sessions with adolescent girls will use Parivartan modules to recognise and examine manifestations of gender disparity and gender-based violence, and empower girls to call for equality and their rights, especially their rights to education and freedom from discrimination. Safe spaces will be arranged for the girls to meet regularly and to nurture their networks. Through these group sessions mentoring will be on issues of violence against girls, sexual and reproductive health education, and developing life skills such as interpersonal negotiation and leadership. These processes will prepare local adolescent support groups to take a lead role in addressing the issues that are of greatest concern to them.

Objectives

Long-Term

- To improve the confidence of adolescent girls
- To improve the accessibility and expand opportunities for girls to enter and continue formal secondary education and the job market

Immediate

- To create awareness of the government schemes/subsidies that encourages their education
- To improve attendance in tutorial classes
- To facilitate participation in Parivartan Plus group sessions
- To increase participation in career counselling to enhance options after school completion
- To support shifts in attitudes and perceived norms among SC/ST girls about the value of completing secondary education for girls and not marrying before age 18

Framework of Expected Outputs, Indicators, and Means of Verification

Outputs – 1	Indicators	Means of Verification	Target
Girls are aware of and linked to schemes that provide assistance and enable them to continue school education	• Number of adolescent girls contacted to discuss about scholarship and schemes<br>• Number of awareness events organised<br>• Number of adolescent girls who attended the awareness programmes on schemes<br>• Number of adolescent girls assisted in accessing these schemes	• Monthly outreach register<br>• Event reports<br>• Monthly reports on social entitlement enrolment	• 3600 adolescent girls contacted<br>• 91 awareness events organised<br>• 80% of adolescent girls attend the programmes<br>• 3600 unique adolescent girls receive entitlements /schemes

Activities

- Linelist the girls and profile them to understand their risk and vulnerability
- Map schemes and subsidies available for the adolescent girls and their families to retain girls in schools
- Create awareness among adolescent girls about these schemes
- Increase demand for these schemes from schools
- Assist adolescent girls to apply for and receive these government subsidies and schemes

Outputs – 2	Indicators	Means of Verification	Target
Tutorial instruction is provided to supplement classroom learning for girls	• Number of tutorial classes organised by the project<br>• Number of adolescent girls who enrolled in the tutorial classes organised by the project<br>• Number of adolescent girls who completed tutorial classes organised by the project	• Monthly reports<br>• Performance mark card of adolescent girls	• 25 tutorial classes organised annually<br>• 720 unique girls during the project and 120 unique girls for each district , every year<br>• 805 of enrolled girls complete classes

Activities

- List girls who require support of tutorials and remedial classes based on the mid-term performance results
- Organise tutorials and remedial classes for the subjects required by the adolescent girls

Outputs – 3	Indicators	Means of Verification	Target
Groups are formed for girls to provide safe space to discuss their issues and build solidarity using the Parivartan curriculum	❖ Number of villages where group sessions are conducted<br>❖ Number of group of adolescent girls formed for Parivartan<br>❖ Number of adolescent girls attending the group sessions on Parivartan<br>❖ Number of groups of adolescent girls that completed Parivartan curriculum<br>❖ Number of adolescent girls who completed the group sessions on Parivartan<br>❖ Number of leadership trainings organised for the peer leaders of groups<br>❖ Number of peer leaders who attended	❖ Group session reports<br>❖ Monthly progress report	❖ 70% of the villages where group sessions will be conducted<br>❖ Each selected village will have 1-2 groups<br>❖ 1800 unique adolescent girls who are presently in high school from the intervention villages attend the Parivartan sessions<br>❖ 100% of groups complete the group sessions<br>❖ 8 batches of leadership training organised<br>❖ 240 girls trained as leaders

Activities

- Finalise Parivartan modules.
- Identify and line list adolescent girls to be part of group reflective sessions
- Identify Parivartan mentors in the villages
- Train mentors on group session Parivartan curriculum
- Organise group sessions for adolescent girls to reflect on and gain skills in reproductive and sexual health, life skills, decision making, etc., using the Parivartan curriculum
- Organise trainings to improve communication and leadership skills with selected girls, especially peer leaders

Output – 4	Indicators	Means of Verification	Target
Girls participate in career counselling sessions and avail options available after completion of high school education	❖ Number of schools that conducted career counselling organised by the project<br>❖ Number of adolescent girls who are attending career counselling sessions organised by the project<br>❖ Number of conventions organised in the district and number of adolescent girls who attended the sessions	❖ Session reports<br>❖ Event report<br>❖ Monthly progress report	❖ 69 schools conduct career counselling<br>❖ All adolescent girls who are studying in 10th standard in 69 high schools receive career counselling<br>❖ One adolescent girl convention per district every six months

Activities

- Identify resource persons to conduct career counselling for class 10 adolescent girls
- Organise career counselling sessions in the schools
- Organise a convention for selected adolescent girls to build solidarity and expand their career options

Key Activities and Timelines

Key activities	Jun-Aug, 2013	Sep-Nov, 2013	Dec-Feb, 2014	Mar-May, 2014	Jun-Aug, 2014	Sep-Nov, 2014	Dec-Feb, 2015	Mar-May, 2015	Jun-Aug, 2015	Sep-Nov, 2015	Dec-Feb, 2016	Mar-May, 2016
Line list the girls and profile them to understand their risk and vulnerability.	■											
Map schemes and subsidies available for adolescent girls and their families.	■											
Educate adolescent girls about these schemes and demand these schemes from schools.		■										
Establish links with Sneha clinics and Santwana clinics and child protection officers.		■										
List girls who require tutorials and remedial classes based on the mid-term performance results.			■									
Organise tutorials and remedial classes for the subjects required by the adolescent girls.			■									
Finalise Parivartan modules.	■											
Identify and line list adolescent girls to be part of group reflective sessions.		■										
Identify Parivartan mentors in the villages.		■										
Train mentors on group session Parivartan curriculum.			■									
Organise group sessions for adolescent girls to reflect on and gain skills in reproductive and sexual health, life skills, decision making, etc., using the Parivartan curriculum.			■	■	■	■	■	■	■	■	■	■
Organise trainings to improve communication and leadership skills with selected girls, especially peer leaders.					■							
Identify resource persons to conduct career counselling for adolescent girls who are studying in class 10.					■							
Organise career counselling sessions in the schools.					■							
Organise a convention for selected adolescent girls to build solidarity and create new options for their future career.				■			■					

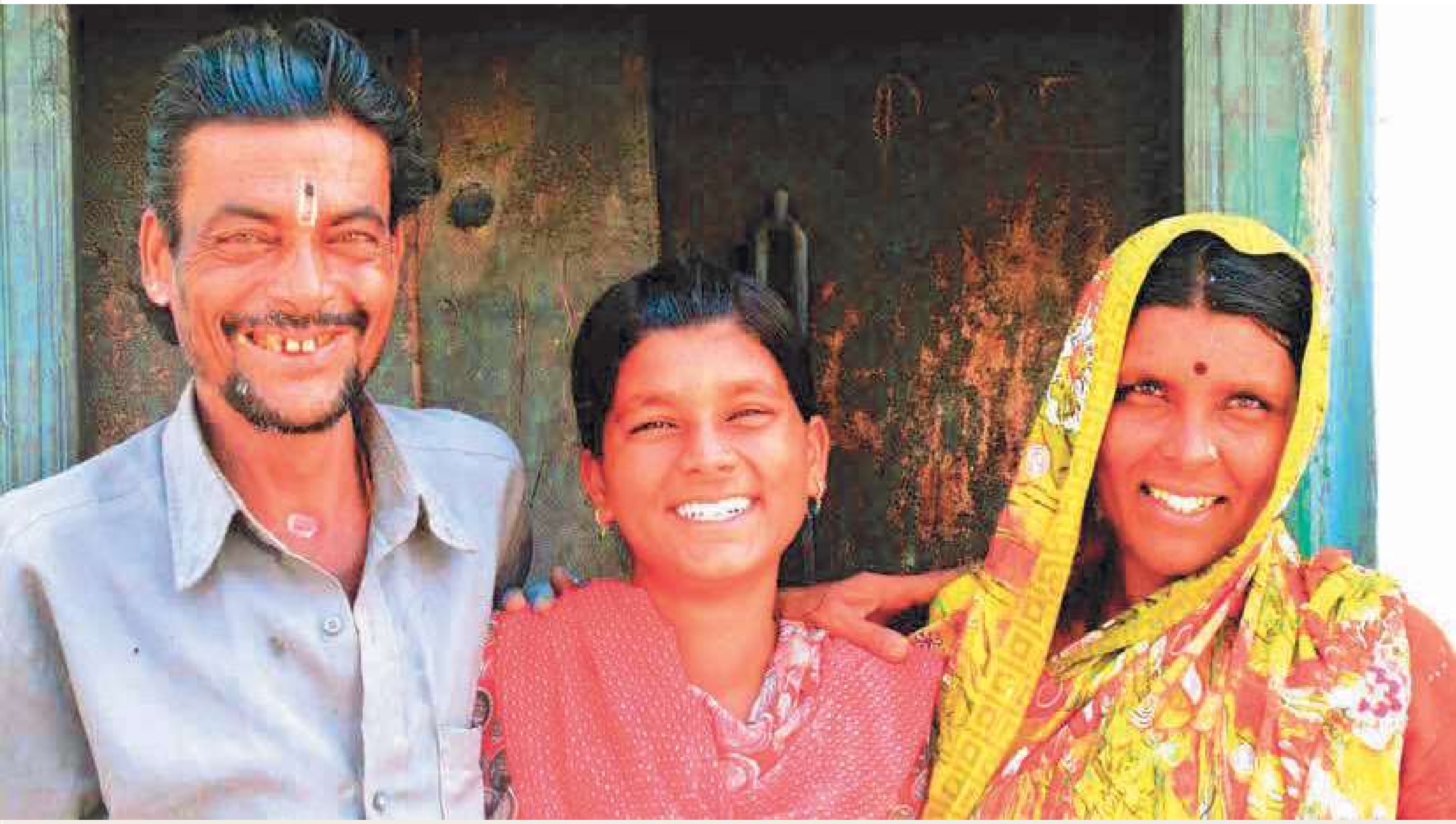

Intervention with Families of Adolescent Girls, including Parents and Decision Makers

Samata will help families to understand the importance of educating girls, the consequences of early marriage and assist them to find ways to afford their daughters' education.

Intervention at the family level intends to create an enabling environment for girls' education by helping families to understand the importance of educating girls and gender equity, and the consequences of early marriage and child bearing, and assisting families to find ways to afford to educate their daughters. This will be done by identifying the most marginalised and vulnerable families, counselling them on the key issues they are facing, helping them solve their problems, initiating dialogue about secondary education for daughters, and linking them to livelihood schemes. These activities will be carried out by the outreach workers through meeting with the parents, village and community-level meetings, samvaada[6] programmes, village-level campaigns, and so on. Intervention with families will also promote their active participation in SDMCs.

[6] Samvaada means dialogue. In Samata intervention villages it is planned to conduct street plays and folk shows on the issues of early marriage, school dropouts, etc. There will be discussions and conversations with the community members on their reactions and views at the end of the show. This is one way of addressing their values and perspectives on their issues and helps the community to reconsider their perspectives and positions. The process of discussions is called Samvaada.

Objectives

Long-Term

- To bring positive changes in gender norms around marriage and education of girls
- To improve accessibility and expand opportunities for girls to enter and continue formal secondary education
- To enhance the engagement and accountability of families for the education of adolescent girls

Immediate

- To support changes in gender norms around marriage and education of girls among families
- To increase engagement and accountability of families for the education of adolescent girls
- To increase awareness on the issues, schemes and options
- To increase participation of family members in campaigns and discussions and problem solving
- To increase the number of families allowing daughters to attend special/tuition class
- To increase utilisation of entitlements/livelihood schemes by families and girls

Framework of Expected Outputs, Indicators, and Means of Verification

Outputs - 1	Indicators	Means of Verification	Target
Family members have greater awareness about the consequences of girls discontinuing education	❖ Number of folk media programmes conducted in the village<br>❖ Number of families who attended the Samvaada programme conducted in the villages<br>❖ Number of meetings held with the community groups and parents<br>❖ Number of vulnerable girls identified during outreach<br>❖ Number of families with vulnerable girls contacted during outreach and counselled	❖ Monthly outreach & activity register of ORWs<br>❖ Event reports<br>❖ Meeting records<br>❖ Documentation from the events	❖ 119 folk media shows conducted<br>❖ 80% of the families that have adolescent girls participate in Samvaada<br>❖ 400 vulnerable girls identified during outreach<br>❖ 400 families met every month and the remaining once every quarter

Activities

- Line list girls and their families, and identify the most vulnerable families
- Conduct folk media performances in the community to initiate dialogue about secondary education for adolescent girls, the hazards of early marriage, early child-bearing, and early sexual debut
- Regular outreach to counsel the most vulnerable families

Outputs- - 2	Indicators	Means of Verification	Target
Parents are aware of and linked to schemes that provide assistance and enable them to send girls to school.	❖ Number of counselling sessions on schemes for parents<br>❖ Number of parents who received counselling on government schemes/ subsidiaries	❖ Monthly outreach & activity register of ORWs<br>❖ Attendance records	❖ 3600 families of adolescent girls in project period of three years<br>❖ At least one from each family

Activities

- Inform families about government schemes that provide material and financial incentives and rewards for educating daughters
- Link families to livelihood or alternative income schemes

Key Activities and Timelines

Key Activities	Jun-Aug, 2013	Sep-Nov, 2013	Dec-Feb, 2014	Mar-May, 2014	Jun-Aug, 2014	Sep-Nov, 2014	Dec-Feb, 2015	Mar-May 2015	Jun-Aug, 2015	Sep-Nov, 2015	Dec-Feb, 2016	Mar-May, 2016
Line list girls and their families and identify the most vulnerable families.												
Conduct folk media performances in the community.												
Educate parents about SDMCs and promote effective parental participation in SDMCs.												
Familiarise families with livelihood and alternative income schemes and facilitate linkages.												

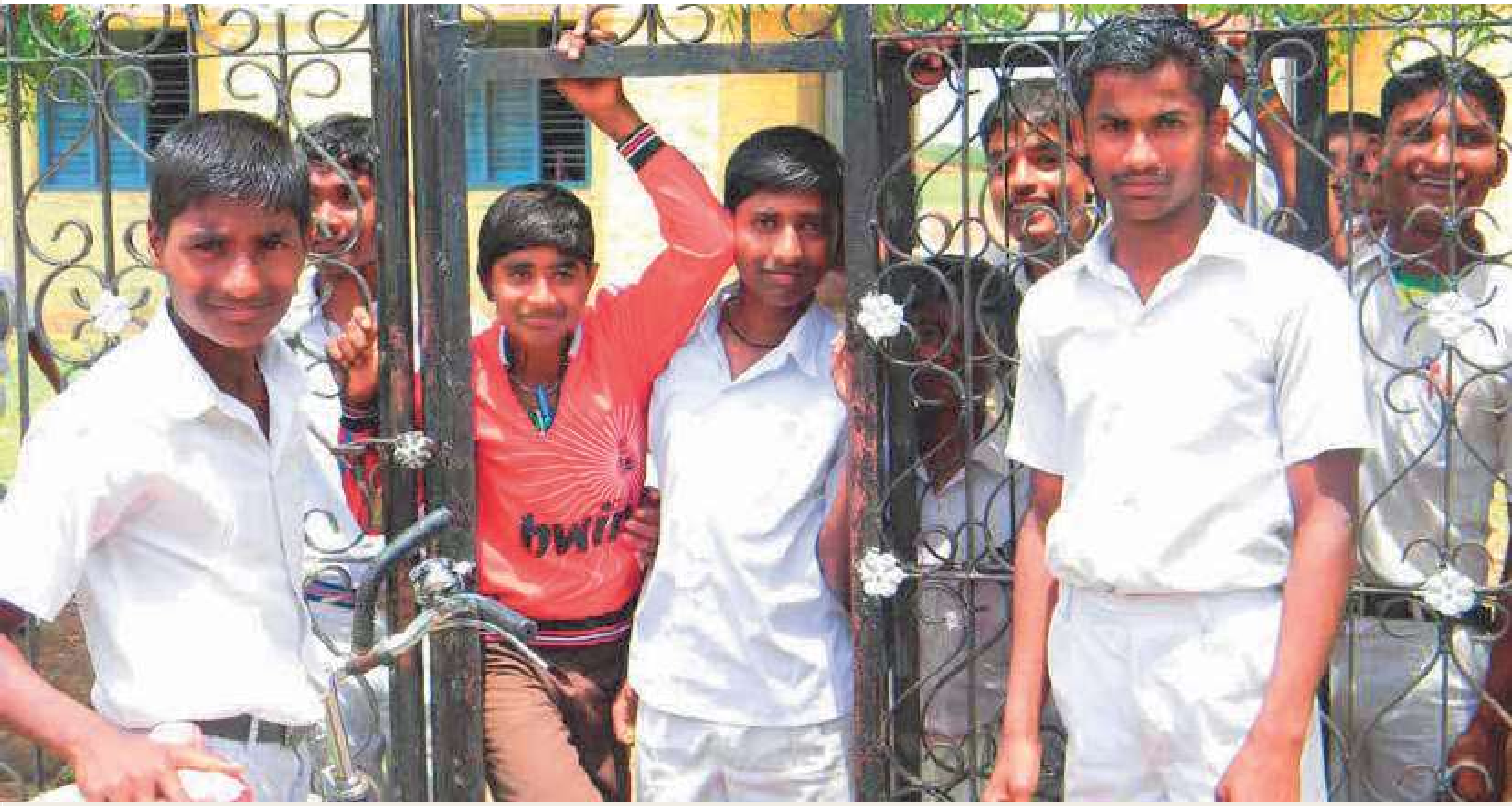

Intervention with Boys (High School Boys 13–18 Years Old from SC and ST Community)

Samata will work with boys to transform their attitudes towards gender, emphasising the right of adolescent girls to a life free of violence and abuse.

The project will work with boys to transform their attitudes towards gender, emphasising the right of adolescent girls to a life free of violence and abuse. It will achieve positive changes in boys' attitudes and behaviours and promote adolescent girls' participation and retention in schools. This will assist girls to enjoy opportunities in education, employment, marriage, and social life on par with boys.

Popular sports will be used as a channel for communicating positive messages on masculinity and respect for women. Samata will use Parivartan, a programme that engages local athletic coaches to deliver violence prevention scripts and tools to adolescent male athletes from the same locality to alter norms that foster aggression and violence, to promote bystander intervention, and to reduce physical and sexual assault. Parivartan uses athletic coaches because they are often seen by boys as role models.

The Parivartan model has been developed and piloted by a team of experts from the International Centre for Research on Women (ICRW) and Futures without Violence along with intervention partners, such as Apnalaya and PATH.

Objectives

Long-Term

- To promote positive change in gender norms around marriage and education of girls
- To enhance the engagement, support, and accountability of boys for the education of the girls

Immediate

- To support change in gender norms around marriage and education of girls
- To enhance engagement, support and accountability of communities for the education of adolescent girls
- To increase participation of boys in campaigns and discussions and problem solving
- To increase vigilance among boys on girl child marriage and school drop out

Framework of Expected Outputs, Indicators, and Means of Verification

Outputs	Indicators	Means of Verification	Target
Forums are formed for boys to reflect on and challenge gender norms using the Parivartan curriculum.	❖ Number of villages where group sessions conducted<br>❖ Number of group of boys formed for Parivartan<br>❖ Number of groups of boys completed Parivartan curriculum<br>❖ Number of boys attending the group sessions on Parivartan<br>❖ Number of boys completed the group sessions on Parivartan curriculum<br>❖ Number of champions identified from the boys	❖ Group session reports<br>❖ Champions register	❖ 70% of villages<br>❖ 270 groups over 3 years<br>❖ 270 groups complete parivartan plus<br>❖ 2730 boys attending and completed group sessions<br>❖ At least 3-4 champions from boys per village

Activities

- Conduct feasibility study on adopting Parivartan model in the north Karnataka context
- Finalize modules of sessions with boys
- Select mentors in the villages to become Parivartan mentors
- Engage boys in critical reflection using the Parivartan curriculum on issues related to gender, sex, sexuality, and violence that affect retention of girls in school through group sessions with boys in their neighbourhood
- Development of champions among family to support girls' education

Key Activities and Timelines

Key Activities	Jun-Aug, 2013	Sep-Nov, 2013	Dec-Feb, 2014	Mar-May,2014	Jun-Aug, 2014	Sep-Nov, 2014	Dec-Feb, 2015	Mar-May, 2015	Jun-Aug, 2015	Sep-Nov, 2015	Dec-Feb, 2016	Mar-May,2016
Develop and finalize modules for group sessions with boys.	■	■										
Identify coaches and mentors.		■										
Conduct group sessions with boys.			■	■	■	■	■	■	■	■	■	■
Identify champions from boys to support the cause of girls.			■									
Train champions.				■	■	■						

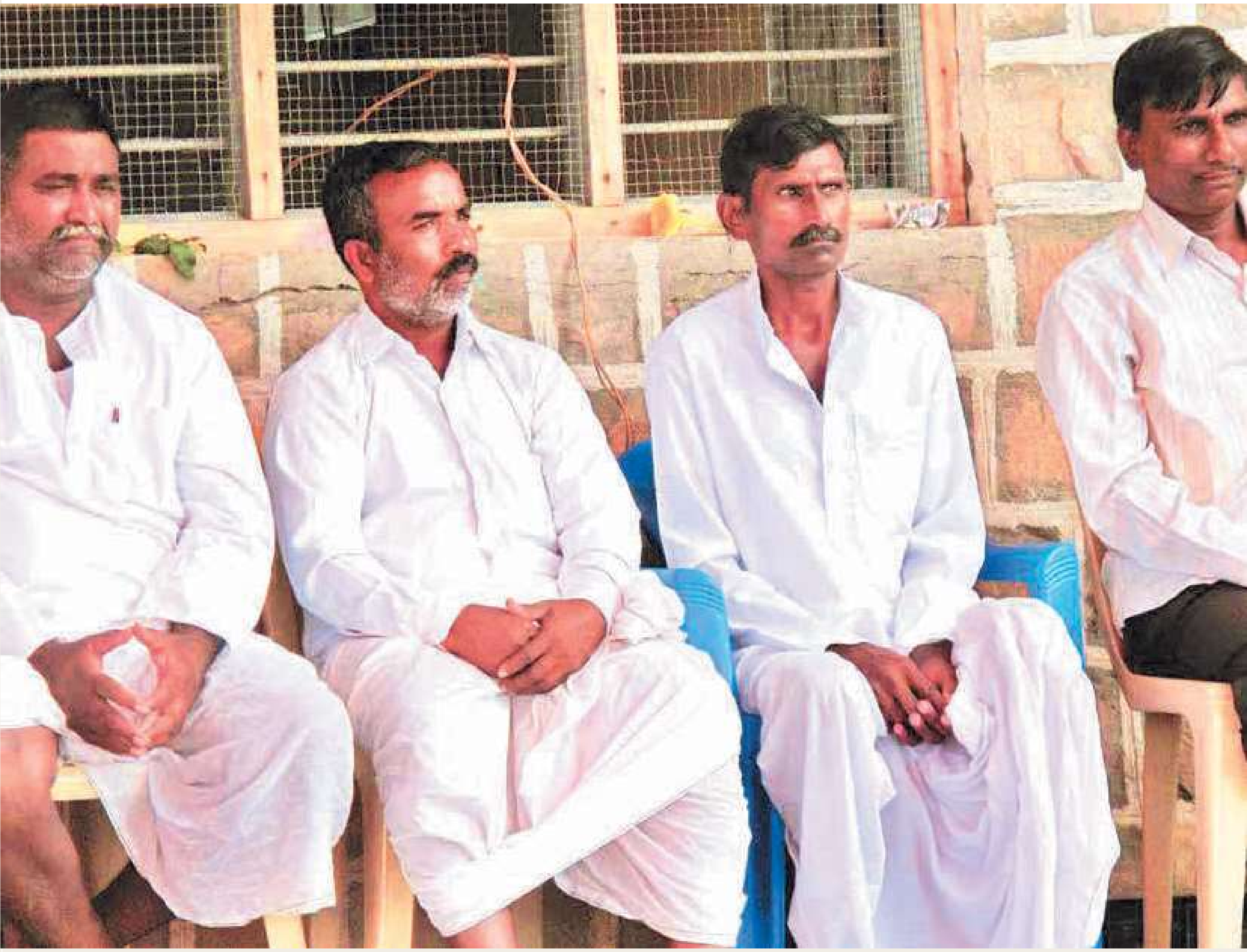

Intervention with Community (Community Leaders, SC/ST Community, Community Groups, such as DSS/Youth Groups, NGOs, and CBOs, in Intervention Villages)

Interventions at the community level aim to increase recognition of the importance of educating girls, gender norms, and the consequences of early marriage, teenage pregnancy, early child bearing, etc., with the objective of building popular support for girls' education. The interventions will be at the village level, involving the key leaders, opinion makers, and influential persons. This is also to build necessary support systems and an environment that is conducive for girls to complete secondary education.

Objectives

Long-Term

- To bring positive change in gender norms around marriage and education of girls among community members
- To enhance engagement, support, and accountability of communities for the education of adolescent girls.

Immediate

- To promote change in gender norms around marriage and education of girls
- To enhance engagement, support and accountability of communities for the education of adolescent girls
- To improve accessibility and expanded opportunities for girls to enter and continue formal secondary education
- To increase awareness about the issue of school drop outs and its negative outcomes, and schemes and options to keep girls in school
- To increase participation of community members in campaigns and discussions and problem solving
- To improve understanding of the barriers and solutions to the issue
- To increase vigilance among community groups on girl child marriage and school drop out

Framework of Expected Outputs, Indicators, and Means of Verification

Output – 1	Indicators	Means of Verification	Target
Community awareness is raised about consequences of girls discontinuing education	❖ Number of folk media programmes conducted in the village and number of community members who participated<br>❖ Number of villages covered with folk media programmes<br>❖ Number of Samvaada programmes conducted in the villages<br>❖ Number of meetings held with the community groups<br>❖ Number of individuals who participated in the community groups	❖ Event report<br>❖ Monthly outreach & Activity Report	❖ One folk media event per year per villager<br>❖ One meeting with the community groups every month in 91 villages<br>❖ 10 members participate in each group meeting

Activities

- Folk media troupes will be selected to develop folk shows on the subjects
- Conduct folk media performances in the community to initiate dialogue about secondary education for adolescent girls, the hazards of early marriage, teenage pregnancy, early child-bearing, and early sexual debut
- Conduct regular meetings with the existing groups in the villages

Output – 2	Indicators	Means of Verification	Target
Community members take action against girl child drop out from schools.	❖ Number of meetings held with the local groups<br>❖ Existence of vigilance committee of the local community<br>❖ Number of meetings held with PRIs on girl child education related issues<br>❖ Mapping of community groups<br>❖ Resource mapping	❖ Advocacy register<br>❖ Meeting reports	❖ One meeting with the community group once in a month<br>❖ One committee for each village<br>❖ One meeting with PRI every quarter on the issue of girl child education

Activities

- Meet regularly with DSS, youth groups, and SHGs to share evidence, progress, and outcomes of the intervention
- Meet regularly with PRIs to help them understand their role in girl child education
- Develop vigilance committees to increase entry and retention
- Support campaigns related to transition and retention started by local community/SDMC/schools
- Advocate with PRIs on the importance of the issue and the need for monitoring the activities undertaken by school

Key Activities and Timelines

Key Activities	Jun-Aug, 2013	Sep-Nov, 2013	Dec-Feb, 2014	Mar-May,2014	Jun-Aug, 2014	Sep-Nov, 2014	Dec-Feb, 2015	Mar-May, 2015	Jun-Aug, 2015	Sep-Nov, 2015	Dec-Feb, 2016	Mar-May,2016
Develop script and identify folk media troupes.												
Conduct folk media performances in the community.												
Meet regularly with DSS, youth groups, and SHGs to share evidence, progress, and outcomes of the intervention.												
Meet regularly with PRIs to help them understand their role in girls' education.												
Form vigilance committees to increase transition and retention.												

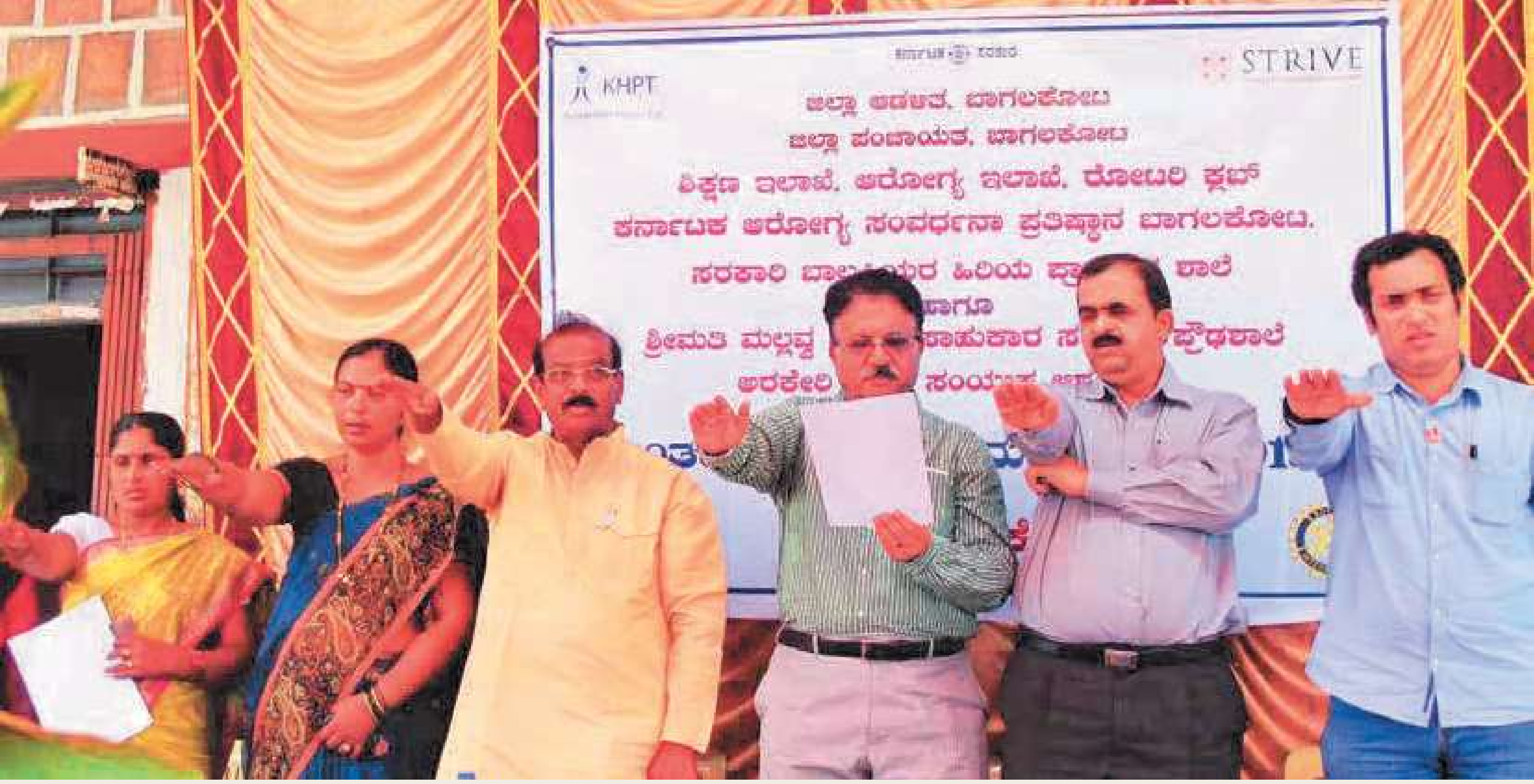

Intervention with State, District, and Block Level Education Department Officials and the Media

Interventions with Education Department officials to increase adolescent girls' educational attainment will be of two types:

i) Interventions that form and strengthen collaboration between the officials, Samata, and civil society

ii) Advocacy

Collaboration with the Education Department is decisive for Samata's successful implementation and large-scale impact because only through collaboration will Samata be able to sensitize officials about adolescent girls' issues and obtain the department's cooperation, imprimatur, and, eventually, ownership. Samata will need the Education Department's imprimatur to ensure the cooperation and participation of school staff in Samata activities. We also must obtain the department's authorization to hold trainings for school staff and SDMCs, and to collect school data and records. Furthermore, it is critical to collaborate closely with the government because government departments control considerable resources for girls' education and welfare.

The ultimate purpose of collaboration with the Education Department is to prepare the government to continue Samata's innovations after KPHT exits. KHPT will work to transition ownership of Samata's innovations to the government by

integrating and conforming them with the government's normative guidelines and developing the government's capacity to scale them up and sustain them across the state.

The present situation in Karnataka is very conducive for this collaboration. Although the government has made several attempts to reduce the dropout rate of adolescent girls from most-marginalised families, school dropout rates at crucial stages of schooling like 5th, 7th, and 10th standards remain high. There are hardly any structural interventions addressing this issue, and the government acknowledges that some of its most effective schemes struggle to scale up [148].

Therefore, the government is seeking support to implement comprehensive programmes for adolescent girls and their education [10, 148]. The government recently invited civil society organizations to contribute ideas for working with adolescent girls and recognises the need for vibrant public participation in the implementation and monitoring of the RTE Act [148]. These circumstances create a very promising opportunity for KHPT to collaborate with the Education Department for Samata's implementation and advocate with the government for the scale-up of Samata's successful interventions.

KHPT's considerable past experience advocating for and assisting the government to scale up programmes has taught us the importance of choosing the right influence strategy at the start of the intervention. When planning an intervention it is critical to develop influence strategies; outline goals and objectives; and define the inputs, outputs, and outcomes, networks of influence and the key audiences so that relationships can then be established and strengthened over the course of the project. We see four important prospects for influencing the government.

Collaboration with the Department of Education is decisive for Samata's successful implementation and to achieve large-scale impact.

One opening for advocacy on adolescent girls' behalf is elected officials' growing recognition of the considerable return on investments in young people, particularly the potential political dividend. Youth and adolescents constitute a major future voting block, and parents are an immense active voting block, so responding to their needs can shape the outcome of elections. Political parties' self-interest renders elected officials receptive to demands from parents and students for political action to improve education for adolescent girls.

Government cells, departments, committees, and schemes for the welfare of children and adolescents are another window of opportunity to advocate with the government for programmes with adolescent girls because these bodies and schemes require

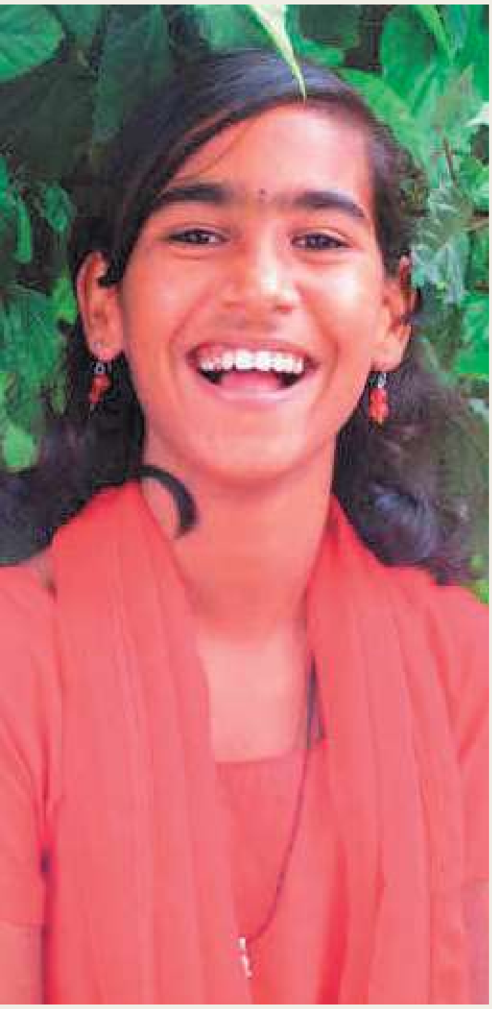

assistance to fulfill their purpose. The SC/ST Departments focus on the development of SC/ST families. In each district there is a Child Welfare Committee comprised of members from civil society. Under the Women and Child Welfare Department there is a Child Protection Cell with a child protection officer. At the state level there is a body called the Child Rights Forum. Efforts through Samata to assist and strengthen these bodies and schemes will be welcomed and appreciated by the government.

The existence of many NGOs and CBOs that work for the cause of adolescent girls and vulnerable women in Samata's project area will strengthen Samata's implementation and provide allies for advocacy. There is considerable experience and expertise within these organisations on adolescent girls' issues, and close communication and coordinated action with these organisations will strengthen advocacy. The organisations include Mahila Samakhya (an NGO working with vulnerable women and children) Janawadi Mahila Sangha (an NGO working with vulnerable women and children), Chaitanya Mahila Sangha (a CBO working with devadasis and other sex workers and their children), Vishala (an NGO working with vulnerable women and children), POWER (an NGO working with vulnerable women and children), FEDINA (an NGO working with vulnerable women and children), St. Anne's (a faith-based NGO working with vulnerable women and children), and district- and taluk-level federations of Sri Shakti women's self-help groups.

Another important ally in advocacy will be the adolescent girls. In the initiatives planned with the adolescent girls, one key strategy is to hold events that enable girls to voice their needs, concerns, and aspirations directly to key stakeholders, like state, district, and block-level officials of government departments, especially the Education Department.

Interventions with the Department of Education will focus on alliance building and collaboration with the government and other stakeholders.

Broadly, the interventions with Education Department officials focus on collaboration and alliance building with key stakeholders, like government, civil society organisations, and networks of nongovernmental organisations. This process includes many activities, including forming contacts at different levels, collaboration activities, joint meetings and conferences, facilitating exposure to programmes, sharing best practices, research and assessment findings, networking with the organisations at different levels, media sensitisation, and direct engagement with the policy makers to translate Samata's learnings into guidelines and schemes.

Objectives

Long-Term

- To ensure that the government develops policies and guidelines based on the outcome of KHPT 's intervention model for scale-up

Immediate

- To increase understanding and support from government towards these interventions

Framework of Expected Outputs, Indicators, and Means of Verification

Outputs - 1	Indicators	Means of Verification	Target
Government supports implementation of the project	❖ Number of circulars issued by the GoK on revision of AG programme guidelines<br>❖ Percentage increase in budget allocation for adolescent girls' programmes<br>❖ Percentage increase in the number of districts where adolescent girls programmes are implemented<br>❖ Number of meetings held with different stakeholders<br>❖ Number of meetings held with civil society organisations	❖ Meeting reports<br>❖ Copy of circulars issued<br>❖ Number of districts where the programme has been scaled up	❖ Meeting with key state officials once in a year<br>❖ Quarterly meeting with district and block level officers

Activities

- Desk review of GoK's existing programmes for adolescent girls and budget allocation details, and prepare a document on the same
- Presentation to GoK officials on the need for scale-up and increased funding for adolescent girls' programmes
- Development of draft policy and guideline document on intervention with adolescent girls.
- Stakeholder consensus-building meeting
- Presentation of draft policy and guidelines on intervention with adolescent girls to GoK officials
- Follow-up meeting with GoK officials on policy adoption and approval
- Organise a meeting of civil society organizations (NGOs/CBOs) working with adolescent girls to pressure GoK to increase funding for adolescent girls programmes
- Follow-up meetings and submissions by civil society organizations to GoK
- Organise policy dialogue meetings with key stakeholders including GoK officials through workshops, seminar, and symposiums

Outputs - 2	Indicators	Means of Verification	Target
Government understands the importance and successful strategies of this intervention	❖ Number of state officials who participated in the programmes<br>❖ Number of programmes<br>❖ Number of circulars and orders issued in support of adolescent programmes	❖ Meeting reports	❖ Half-yearly reviews with state officials and quarterly reviews with district and block level officers

Activities

- Establish key contacts at the state, district, and block levels and regularly update them on the progress and outcomes of the project
- Organise meetings and conferences to share evidence on the issue at district level
- Participate in important government meetings on adolescent girls and education
- Organise field visits for government officials to the intervention sites
- Share reports of the project
- Prepare and document success stories
- Hold advocacy meetings at district and state level
- Network with organisations working with adolescent girls, including education, in Karnataka and outside
- Support GoK to adapt Samata's lessons in guidelines and schemes for entry and retention

Outputs - 3	Indicators	Means of Verification	Target
Media coverage	❖ Number of articles and radio or televised broadcasts	❖ Article clippings<br>❖ Webpage URLs<br>❖ Recordings of broadcasts	❖ 12 clippings and one recording

Activities

- Facilitate media coverage of positive AG stories and the evidence emerging from the project
- Sensitise selected media reporters on the issue of AGs
- Use media to cover positive messages about the programme and activities being implemented under the programme
- Share best practices with the media for wider coverage in the media
- Organise interviews with AGs, teachers, and families

Key Activities and Timelines

Key Activities	Jun-Aug, 2013	Sep-Nov, 2013	Dec-Feb, 2014	Mar-May, 2014	Jun-Aug, 2014	Sep-Nov, 2014	Dec-Feb, 2015	Mar-May, 2015	Jun-Aug, 2015	Sep-Nov, 2015	Dec-Feb, 2016	Mar-May, 2016
Conduct a desk review of GoK's existing programmes for adolescent girls and budget allocation details, and prepare a document on the same.	■	■										
Presentation to GoK officials on the need for scale-up and increased funding for adolescent girls programmes.		■										
Develop draft policy and guideline document on intervention with adolescent girls.							■					
Hold stakeholder consensus-building meeting.			■	■	■	■						
Present draft policy and guidelines on intervention with adolescent girls to GoK officials.										■	■	■
Organise a meeting of civil society organisations (NGOs/CBOs) working with adolescent girls to pressure GoK to increase funding for adolescent girls' programmes.		■			■		■					
Follow up meetings and submissions by civil society organisations to GoK.			■			■		■		■		■
Organise policy dialogue meetings with key stakeholders, including GoK officials, through workshops, seminars, and symposiums.											■	
Establish key contacts at the state, district, and block level, and regularly update them on the progress and outcomes of the project.			■			■		■	■	■	■	■
Organise meetings and conferences to share evidence on the issue at the district level.		■		■		■				■		
Organise field visits for government officials to intervention sites.						■		■		■		■
Prepare and document success stories.		■	■	■	■	■	■	■	■	■	■	■
Hold advocacy meetings at district and state levels.		■	■	■	■	■	■	■	■	■	■	■
Network with organisations working with adolescent girls', including education, in Karnataka and outside.						■		■		■		
Facilitate media coverage of positive AG stories and Samata's impact and lessons.					■			■		■		■
