# Supplementary material for: The Samata intervention to increase secondary school completion and reduce child marriage among adolescent girls: results from a cluster-randomised control trial in India
Source: J Glob Health. 2019 Jun 25;9(1):010430. doi: 10.7189/jogh.09.010430 (PMC6684866; doi:10.7189/jogh.09.010430)
Supplement: Online Supplementary Document [file jogh-09-010430-s001.zip › 3_Figure S1 Landscape mapping.pdf]

Contextual timeline

The Samata trial coincided with a range of national, state and district level government programmes aiming to improve girls' education and overall quality of life.

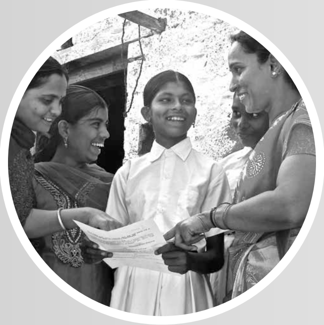

2011–13

Sabla girls' empowerment and life skills programme in Bijapur District; Government of India, implemented by Karnataka state government, Department of Women and Child Welfare; continued in some villages by NGOs

2014–15

Career counselling: Government of India guidelines through National Council of Educational Research and Training (NCERT); implemented in Samata districts (Bijapur and Bagalkot) following district-level circulars, likely in response to positive responses by students in Samata intervention schools

2015

- ***Beti bachao beti Padhao* programme**: Save your daughter, educate your daughter; Government of India
- **Sneha Clinics** (sexual and reproductive health) introduced at school-level

2016–17

- **Cash incentives** for girls to prevent school drop out
- **Remedial classes** (Mission 100) for low-performing students; Karnataka state government

2016–17

School Safety Committees, Karnataka State Department of Education circular

Samata timeline

Girls were enrolled in two cohort waves, one academic year apart. Secondary school starts in year 8.

Cohort 1 exposed to 18 months of intervention activities – starting in year 9.

Cohort 2 exposed to 30 months of intervention activities – starting in year 8.

Endline surveys conducted at the end of year 10 (end of secondary school).

COHORT 1 INTERVENTION DURATION (2014 JUL-SEP to 2015 OCT-DEC)

	2013				2014				2015				2016				2017		
	JAN-MAR	APR-JUN	JUL-SEP	OCT-DEC	JAN-MAR	APR-JUN	JUL-SEP	OCT-DEC	JAN-MAR	APR-JUN	JUL-SEP	OCT-DEC	JAN-MAR	APR-JUN	JUL-SEP	OCT-DEC	JAN-MAR	APR-JUN	JUL-SEP
**Cohort 1:** Girls leaving primary school in 2013		Finish primary school in year 7	Enrol in secondary school year 8		Trial start survey		Samata activities start<br>Enrol in year 9				Enrol in year 10		Year 10 exams	Trial end survey					
**Cohort 2:** Girls leaving primary school in 2014						Finish primary school in year 7	Samata activities start<br>Enrol in secondary school year 8	Trial start survey			Enrol in year 9				Enrol in year 10		Year 10 exams	Trial end survey	

COHORT 2 INTERVENTION DURATION (2014 JUL-SEP to 2016 OCT-DEC)
