# Supplementary figures and images for: The Samata intervention to increase secondary school completion and reduce child marriage among adolescent girls: results from a cluster-randomised control trial in India
Source: J Glob Health. 2019 Jun 25;9(1):010430. doi: 10.7189/jogh.09.010430 (PMC6684866; doi:10.7189/jogh.09.010430)

# Samata Theory of Change

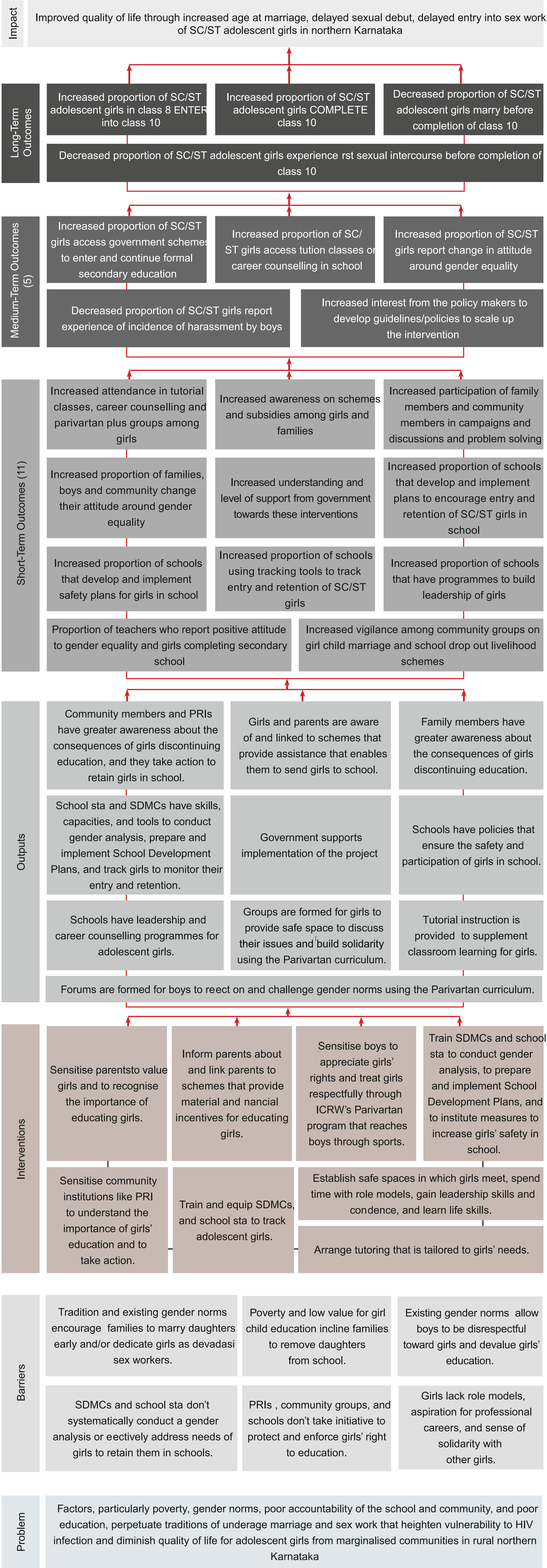

Supplement: Online Supplementary Document [file jogh-09-010430-s001.zip › 4_Fig S2 Project ToC.pdf]
